# Elucidating the regulatory mechanism of Swi1 prion in global transcription and stress responses

**DOI:** 10.1038/s41598-020-77993-0

**Published:** 2020-12-14

**Authors:** Zhiqiang Du, Jeniece Regan, Elizabeth Bartom, Wei-Sheng Wu, Li Zhang, Dustin Kenneth Goncharoff, Liming Li

**Affiliations:** 1 Department of Biochemistry and Molecular Genetics, Northwestern University, Chicago, 60011 USA; 2 Department of Electrical Engineering, National Cheng Kung University, Tainan City, 701 Taiwan; 3 Chinese Institute for Brain Research, Genomics Center and HPC Core, Beijing, 102206 China

**Keywords:** Prions, RNA sequencing

## Abstract

Transcriptional regulators are prevalent among identified prions in *Saccharomyces cerevisiae*, however, it is unclear how prions affect genome-wide transcription*.* We show here that the prion ([*SWI*^+^]) and mutant (*swi1∆)* forms of Swi1, a subunit of the SWI/SNF chromatin-remodeling complex, confer dramatically distinct transcriptomic profiles. In [*SWI*^+^] cells, genes encoding for 34 transcription factors (TFs) and 24 Swi1-interacting proteins can undergo transcriptional modifications. Several TFs show enhanced aggregation in [*SWI*^+^] cells. Further analyses suggest that such alterations are key factors in specifying the transcriptomic signatures of [*SWI*^+^] cells. Interestingly, *swi1∆* and [*SWI*^+^] impose distinct and oftentimes opposite effects on cellular functions. Translation-associated activities, in particular, are significantly reduced in *swi1∆* cells. Although both *swi1∆* and [*SWI*^+^] cells are similarly sensitive to thermal, osmotic and drought stresses, harmful, neutral or beneficial effects were observed for a panel of tested chemical stressors. Further analyses suggest that the environmental stress response (ESR) is mechanistically different between *swi1∆* and [*SWI*^+^] cells—stress-inducible ESR (iESR) are repressed by [*SWI*^+^] but unchanged by *swi1∆* while stress-repressible ESR (rESR) are induced by [*SWI*^+^] but repressed by *swi1∆*. Our work thus demonstrates primarily gain-of-function outcomes through transcriptomic modifications by [*SWI*^+^] and highlights a prion-mediated regulation of transcription and phenotypes in yeast.

## Introduction

The term “prions” was first used to describe the proteinaceous pathogen of the fatal neurodegenerative diseases known as transmissible spongiform encephalopathies (TSEs) or prion diseases^1^. This protein-only concept has now been extended to explain numerous biological phenomena that are conferred by altered, transmissible conformations of otherwise normal host proteins. Indeed, prion and prion-like proteins have now been identified in diverse organisms—virus^2^, bacterium^3,4^, fungus^5^, fruit fly^6^, sea slug^7^, plant^8^, and mammal^9^. Interestingly, there are more than ten prions identified from the budding yeast *Saccharomyces cerevisiae*, which are transmitted as epigenetic elements associated with altered phenotypes^10–13^*.* A large portion of these yeast prion proteins are transcription factors, suggesting that prions may play a role in transcriptional regulation^14–18^.

One yeast prion, [*SWI*^+^], was identified in our laboratory. The protein determinant of [*SWI*^+^] is Swi1, a subunit of the ATP-dependent chromatin remodeling complex SWI/SNF^16^. The SWI/SNF function is evolutionarily conserved from yeast to human and at least seven different subunits of the human SWI/SNF complexes are frequently mutated in ~ 20% of primary tumors across all cancer types^19^. Using energy from ATP hydrolysis, the SWI/SNF complex modulates chromatin conformation via nucleosome sliding or eviction of histones and thus regulate transcription^20–22^. SWI/SNF alone can nonspecifically bind to DNA and is targeted to specific genes by interacting with distinct transcription factors and particular histone modification marks such as H3K4 acetylation^23,24^. Recent studies show that the yeast SWI/SNF can form modular subcomplexes and each module has specific effects on the structure and function of the complex^25,26^.

The yeast Swi1 is a large protein. Its amino-terminal region enriched in asparagine is dispensable for the remodeling function and contains an amyloid core domain that can form amyloid in vitro^27,28^*.* Swi1 harbors a prion domain (PrD) at its N-terminus and the first 30 or so residues is sufficient to support the prion propagation^29,30^. The C-terminal region is not involved in the prion formation or transmission but is essential for normal function of Swi1^27,31^. The middle glutamine-rich region of Swi1 embraces the conserved AT-rich interaction domain (ARID) for DNA-binding and modulates the activities of the N-terminal and C-terminal regions^27^. [*SWI*^+^] relies on molecular chaperones for its propagation^16^, and is sensitive to changes of Hsp70 chaperone activity^32^. The presence of another prion element can either promote or destabilize the Swi1 prion, and vice versa, and the Swi1 prion can also influence the formation and propagation of other prions^33–35^. Swi1 is essential for a number of important cellular functions, such as sporulation, carbon metabolism, and mating type switching^20,36^. Depending on the trait examined, [*SWI*^+^] cells exhibit either partial Swi1 loss-of-function phenotypes, such as non-glucose sugar utilization^16^, or complete loss-of-function phenotypes, such as flocculin gene expression and multicellular features^31^. It has been shown that the complete loss-of-function of Swi1 in [*SWI*^+^] cells is a consequence of both decreased Swi1 function and sequestration of additional Q/N-rich transcriptional activators by the Swi1 prion aggregates^31^. Based on the tight regulation of *FLO* genes by [*SWI*^+^], a reliable [*SWI*^+^] reporter has been developed and used in high throughput screening for anti-prion compound identification^37^. Additional Swi1 prion research is discussed in reviews^38–41^.

Despite our increased knowledge of yeast prion biology, how a transcriptional modulator-based prion alters genome-wide transcription and how such alterations impact cellular functions and phenotypes is poorly understood. In this study, we examined the transcriptional profiles of a set of isogenic S288C strains—wild-type non-prion (wt hereafter), [*SWI*^+^], and *swi1∆* cultivated in a rich medium. Our results show dramatic differences between the *swi1∆* and prion strains. Combining computational analysis and experimental examination, we further explored the mechanism underlying such distinctions and compared the phenotypic difference between *swi1∆* and [*SWI*^+^] cells, particularly in their responses to different stresses.

## Results

### Distinct transcriptomic profiles of [***SWI***^+^] and ***swi1∆*** cells

To examine how [*SWI*^+^] and *swi1∆* differentially affect genome-wide gene expression, we performed RNA-seq experiments to compare the transcriptomes of a set of isogenic S288C strains—wt, [*SWI*^+^], and *swi1∆*. All three strains contain a repaired, and thus, functional *FLO8* gene that is required for expression of flocculin genes and exhibition of multicellular features^31^. Prior to RNA-seq experiments, we confirmed the prion statuses of Swi1 and Rnq1. Rnq1 is the protein determinant of [*PIN*^+^]^42^, also known as [*RNQ*^+^]^43^. The prion status of Rnq1 was examined because de novo formation of [*RNQ*^+^] can be induced by [*SWI*^+^]^35^. As shown in (Fig. S1a), the diffused fluorescent patterns of Rnq1GFP in all three strains indicate that none of them contained the [*RNQ*^+^] prion, excluding the interference of [*RNQ*^+^] to our RNA-seq results. The Swi1 prion state was verified by the aggregation status of Swi1-NQ-YFP, reduced ability to use non-glucose carbon sources, and lack of adhesive growth^16,31^ (Fig. S1a,b). Total RNAs were isolated from triplicated samples and cDNA libraries were constructed after oligo dT-enrichment. Upon sequencing and aligning the reads to a reference yeast genome (S288C strain), we found that one sample of the [*SWI*^+^] strain and one sample of the *swi1∆* strain clustered a little more apart from the other two replicates though all triplicates of the three strains still visibly grouped together (Fig. S1c). Likely, the prion may have been partially lost for the departed [*SWI*^+^] sample due to instability of [*SWI*^+^]^32^ and genetic alterations may have occurred for the departed *swi1∆* sample as reported previously^44^ during cultivation. To ensure the robustness of the sequencing quality, data from the two departed samples were excluded from our analyses. As shown in Fig. S1d, ~ 15–18 million reads, comparable to that of approximate 62–75 × yeast genome size, were collected for each sample and 97–99% of the reads were uniquely mapped to the genome, indicating a good quality of the sequencing. The included yeast isolates do not show ploidy changes (Fig. S1e). The sequencing quality is also justified by confirming that several previously reported genes including *FLO1* and *FLO11* that are downregulated in *swi1∆* and [*SWI*^+^] cells^31^ were indeed similarly downregulated as shown in Fig. S2. These data support the trustworthiness of our RNA-seq results.

**Figure 1:**
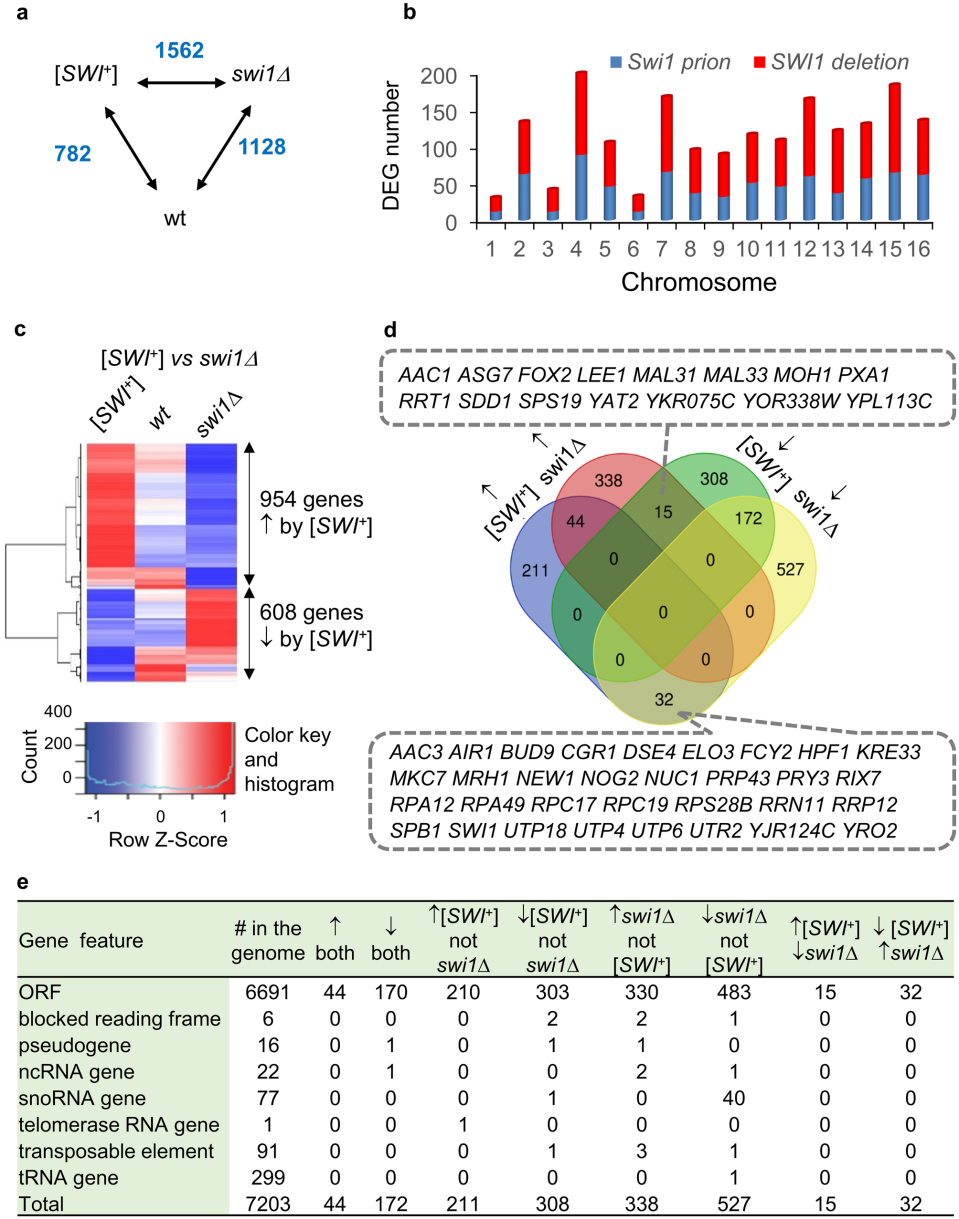
Analyses of [*SWI*^+^]—specific transcriptome. (**a**) Shown are numbers of differentially expressed genes (DEGs) among isogenic [*SWI*^+^], *swi1∆* and non-prion wild-type (wt) strains with a cutoff criteria of Adj. p-value < 0.001 and |log2(FC)|> 1. (**b**) Numbers of DEGs that distribute to each chromosome are plotted. Note, DEGs of [*SWI*^+^] and *swi1∆* strains were acquired upon normalizing to the transcript levels of the wt strain. (**c**) Heatmaps of DEG counts (normalized to FPKM - fragments per kilobase of transcript per million mapped reads ) were obtained by comparing the transcriptomes of the [*SWI*^+^] and *swi1∆* strains. Reads for the wt strain are also included as a reference. (**d**) A Venn diagram illustrating the overlay of DEGs that are either activated or inhibited in the [*SWI*^+^] and *swi1∆* strains when compared to the *wt* strain. Numbers of DEGs are displayed for each regulatory pattern. Genes listed in the dotted boxes showing opposite regulatory patterns in [*SWI*^+^] and *swi1∆* cells: upper, genes that are downregulated in [*SWI*^+^] cells but upregulated in *swi1∆* cells; lower, genes that are upregulated in [*SWI*^+^] cells but downregulated in *swi1∆* cells. (**e**) DEGs collected from [*SWI*^+^] and *swi1∆* cells can be categorized into 8 subgroups according to their transcriptional patterns relative to the wt and can be designated to distinct gene features. Numbers in the table are counts of genes. ↑, activation; ↓, inhibition.

The cutoff for calling differently expressed genes (DEGs) in this study was a fold-change > 2 and an adjusted p-value < 0.001. Using such a criterion, 782 and 1128 DEGs were collected for [*SWI*^+^] and *swi1∆* strains compared to wt, respectively (Fig. 1a, also see Table S1 for raw sequencing data). These DEGs distribute evenly to every chromosome without noticeable biases (Fig. 1b). Surprisingly, [*SWI*^+^] and *swi1∆* strains only share 263 DEGs—23.3% of *swi1∆* DEGs or 33.6% of [*SWI*^+^] DEGs (Table S1 and data not shown). Consistent with this notion, we observed 1562 DEGs (954 are activated and 608 are inhibited by [*SWI*^+^]) when a pairwise comparison of [*SWI*^+^] and *swi1∆* strains was conducted (Fig. 1a,c).

We then performed a detailed analysis of the identified DEGs of [*SWI*^+^] and *swi1∆* obtained by comparing to the wt transcriptome (Fig. 1d). A total of 287 upregulated and 495 downregulated genes were found for [*SWI*^+^] cells, whereas 397 upregulated and 731 downregulated genes were found for *swi1∆* cells, demonstrating that the deletion imposes a greater impact on the yeast transcriptome than [*SWI*^+^] (Fig. 1d and data not shown). Combining DEGs of [*SWI*^+^] and *swi1∆*, we observed a total of 1647 individual DEGs. These genes can be grouped into 8 subgroups based on their regulatory patterns by [*SWI*^+^] and/or *swi1∆* (Fig. 1d), individual DEGs in each subgroup are dispersed evenly across the genome without obvious hotspots or biases for specific DNA regions (Fig. S3). Further investigation revealed that most of the DEG-transcripts are protein coding regions—open reading frames (ORFs) (Fig. 1e). In this regard, among the total 6 blocked ORFs in S288C genome, *YIL167W* and *YIR043C* are activated but *CCW22* is repressed by *swi1∆* while *YDL153C* and *CRS5* are repressed by [*SWI*^+^]. For the 16 pseudogenes in S288C genome, the transcription of *YLL017W* is inhibited by both [*SWI*^+^] and *swi1∆*, *YLL016W* is inhibited only by [*SWI*^+^], and *YIL168W* is activated by *swi1∆*. As a note, even though it’s not the case for S288C-derived strains, *YIL167W* and *YIL168W* may constitute a functional ORF expressing Sdl1—L-serine dehydratase for other yeast strains^45^. Similarly, *YLL017W* and *YLL016W* may form a functional ORF to express Sdc25, an analog of Cdc25 in other yeast strains^46^. Among the total 22 ncRNA genes, *PWR1* is downregulated in both [*SWI*^+^] and *swi1∆* strains, and *RUF20* is downregulated while *HRA1* and *ICR1* are upregulated in the *swi1∆* strain. As the only telomerase gene in the genome, *TLC1* expression is upregulated solely in [*SWI*^+^] cells. Within 91 transposable element genes in the yeast genome, the transcription of *YDR210W-B* is inhibited by *swi1∆, YGR109W-B* is inhibited by [*SWI*^+^], and three other transposable genes, *DR170W-A, YNL284C-B,* and *YDR261W-B* are activated in *swi1∆* cells. Among the total 299 tRNA genes, *tD(GUC)O* is the only one that is merely downregulated by *swi1∆*. In contrast, the regulation of small nucleolar RNA (snoRNA) genes is the most dramatic. Among the 77 snoRNA genes, [*SWI*^+^] only inhibits the expression of *SNR70*, but *swi1∆* downregulates 40 snoRNA genes (Fig. 1e). Taken together, we show that instead of simply mimicking *swi1∆*, [*SWI*^+^] exhibits a unique expression fingerprint distinct from that of wt and *swi1∆*. Our data also suggest modular effects on phenotypes for the two types of cells, which were further analyzed later.

### Contribution of altered expression of TF genes to the observed DEGs

**Figure 2:**
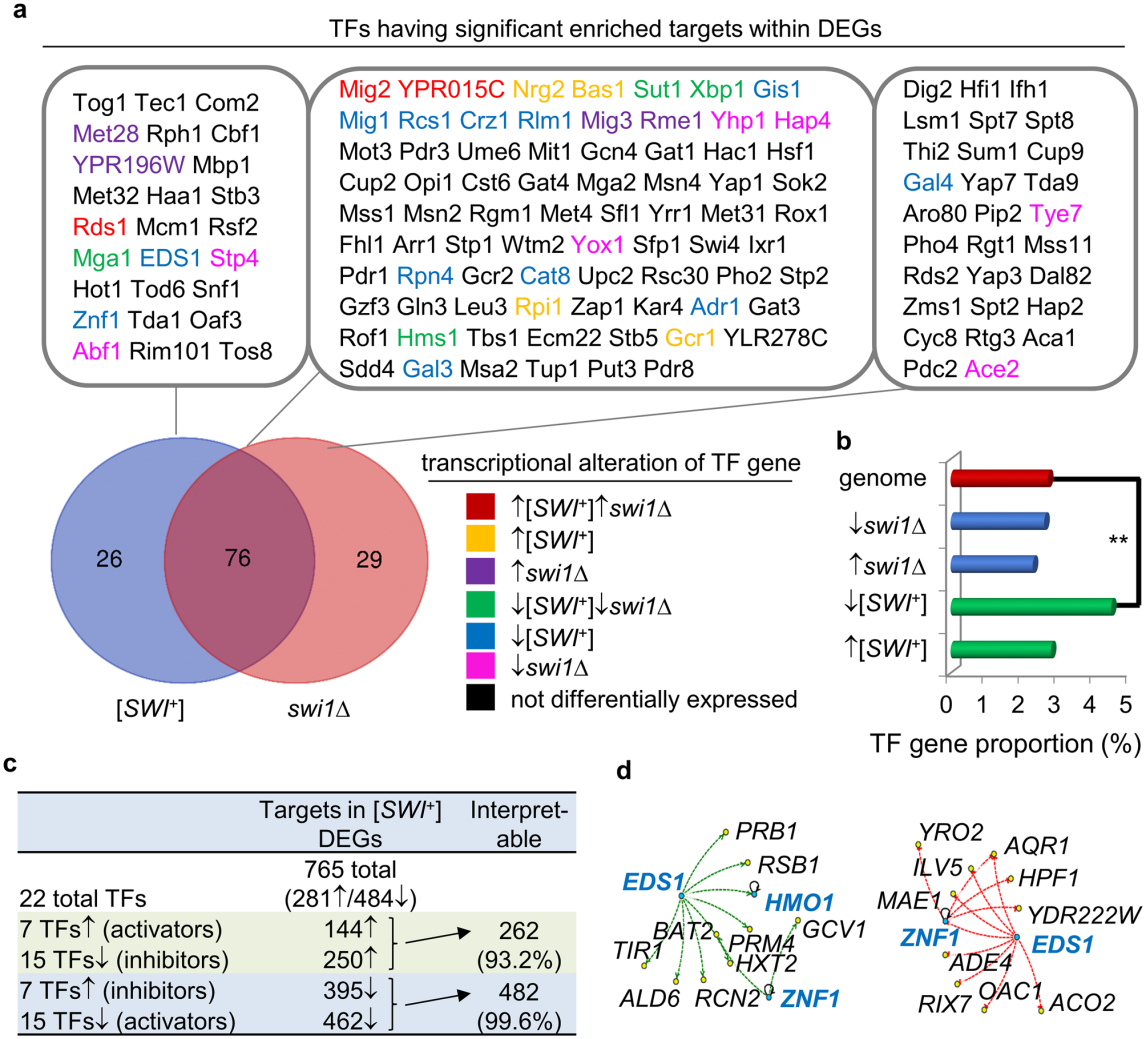
Contribution of altered transcription of TF genes to [*SWI*^+^] DEGs. (**a**) Shown are transcription factors (TFs) that have overrepresented regulatory targets within the DEGs in [*SWI*^+^] and *swi1∆* cells (genes listed in the order of p-value, small to large). TF genes that are regulated similarly or differently by [*SWI*^+^] and *swi1∆* are indicated by different colors, and the overlay of these TFs of [*SWI*^+^] and *swi1∆* are also shown. (**b**) Proportion of upregulated and downregulated TF genes among DEGs in [*SWI*^+^] and *swi1∆* cells. The analysis was based on 183 consensus TF genes defined by YEASTRACT. Significance was calculated using Fisher’s Exact Test that gave a p-value of 0.006. (**c**) 22 TFs with altered transcription in [*SWI*^+^] cells and with overrepresented targets among the prion DEGs (p-value < 0.001 using YEASTRACT, total 765 collective target DEGs). The upregulation of ~ 93.2% (262 combined unique genes out of the 281 total) DEG targets, and the downregulation of ~ 99.6% (482 unique genes out of the 484 total) are interpreted by a combined effect of the transcriptional alterations (up-or down-regulation) and regulatory activities (as inhibitors or activators) of the TFs. The TF/targets associations were defined based on published genetic data according to YEASTRACT. (**d**) Regulatory networks of two overrepresented transcription factors (Znf1 and Eds1) that are downregulated specifically in [*SWI*^+^] cells. Left, DEGs/targets inhibited in [*SWI*^+^] cells when Znf1 and Eds1 function as activators; and right, DEGs/targets activated in [*SWI*^+^] cells when they function as inhibitors. All TFs are in blue.

We speculated that multiple factors may have contributed to the observed differences between wt, [*SWI*^+^], and *swi1∆* transcriptomes, such as changes in the integrity and functionality of the SWI/SNF complex, altered interactions between TFs and SWI/SNF, and DNA and histone modifications. Particularly, the expression and functional alterations of TFs may contribute to the observed DEGs in [*SWI*^+^] and *swi1∆* cells. To test this possibility, we first examined whether target genes of TFs (defined by documented expression evidence) are overrepresented within DEGs in [*SWI*^+^] and *swi1∆* cells. Using YEASTRACT, with a cutoff of p-value < 0.001, we found that 778 potential targets of 102 TFs are among the [*SWI*^+^] DEGs, confirming that TF targets are indeed significantly overrepresented in [*SWI*^+^] DEGs (Fig. 2a, also see Table S2 for details). Similarly, targets of 105 TFs are also significantly overrepresented in the *swi1∆* DEGs, with about 1106 such DEGs being potential targets of these TFs. Among these TFs, 76 are shared by both [*SWI*^+^] and *swi1∆* strains (Fig. 2a). The large number of TFs and their targets found in this analysis was unexpected. One possible explanation is that the recruitment of the SWI/SNF complex to its target promoters requires coordinated actions of certain TFs, and thus Swi1 regulated genes are often TF targets^47,48^.

Nevertheless, the above findings encouraged us to search for individual TF genes that are transcriptionally regulated by [*SWI*^+^] or *swi1∆*, and as a result, we found 34 and 31 such TF genes among DEGs from [*SWI*^+^] or *swi1∆* cells, respectively (Table S2). Further analysis suggests that TF genes are more significantly overrepresented within the prion DEGs, particularly DEGs downregulated by [*SWI*^+^] (Fig. 2b). Interestingly, for TFs having overrepresented targets within [*SWI*^+^] DEGs, 22 of them actually are transcriptionally modified by the prion, including 7 upregulated (*MIG2, YPR015C, RDS1, BAS1, NRG2, RPI1, GCR1*) and 15 downregulated (*SUT1, XBP1, HMS1, MGA1, CRZ1, GIS1, MIG1, RCS1, RLM1, RPN4, CAT8, ADR1, EDS1, GAL3, ZNF1*) TF genes (Fig. 2a and Table S2). Notably, *MIG2, YPR015C, RDS1* are upregulated and *SUT1, XBP1, HMS1, MGA1* are downregulated similarly in *swi1∆* cells. 765 targets of the 22 TFs were identified from the [*SWI*^+^] DEGs—281 activated and 484 inhibited (Fig. 2c, also see regulatory details in Fig. S4 and Table S2). As activators, the upregulation of 7 TF genes can explain the enhanced transcription of 144 DEGs; and as inhibitors, their upregulation can explain the downregulation of 395 DEGs in [*SWI*^+^] cells. Similarly, the upregulation of 250 genes and downregulation of 462 genes in [*SWI*^+^] cells are interpretable by activation and inhibition activities of the 15 downregulated TFs, respectively (Fig. 2c). After removal of the redundant targets, the activation of 262 DEGs (93.2% out of the 281 total upregulated DEG targets) and the inhibition of 482 DEGs (99.6% of the total 484 downregulated DEG targets) are interpretable by transcriptional alterations of the 22 TF genes (Fig. 2c). The interpretable percentage is not 100%, perhaps due to that some DEGs can be regulated by multiple TFs that can act as activators or inhibitors depending on their targets. For example, *Bas1* was predicted to be one of the most versatile upregulated TFs, which can explain the downregulation of 327 DEGs to which it serves as an inhibitor and for the upregulation of 96 DEGs to which it functions as an activator (Table 1 and Table S2). In contrast, *Rnp4* is one of the key TFs that is downregulated in [*SWI*^+^] cells and can be used to explain the downregulation of 395 DEGs and the upregulation of 222 DEGs (Table 1 and Table S2) through activation and inhibition, respectively. The combined actions of Bas1 and Rnp4 may also alter the regulatory output of some targets co-regulated by the two TFs. Interestingly, *EDS1* and *ZNF1* are downregulated and were predicted having overrepresented targets uniquely in [*SWI*^+^] but not in *swi1∆* cells. As shown in Fig. 2d and Table 1, changes in their expression in [*SWI*^+^] cells might explain the upregulation of 10 genes and the downregulation of the other 10.

**Table 1:** 22 [*SWI*^+^]-regulated TFs and their targets.

TF	Target enrichment			Regulation		Targets among [*SWI*^+^] DEGs			
	^#^for [*SWI*^+^]			*swi1∆*	[*SWI*^+^]	Total	Interpretable		
	User (%)	CK (%)	P-value				↑	↓	Ratio (%)
Bas1	66.97	17.39	0		↑	521	96	327	81.2
Mig2	12.21	31.46	0	↑	↑	95	17	65	86.3
Nrg2	5.78	39.13	0		↑	45	0	40	88.9
YPR015C	12.34	25.81	0	↑	↑	96	5	41	47.9
Rpi1	6.30	28.16	4.5E−11		↑	49	7	34	83.7
Rds1	6.81	20.31	1.9E−06	↑	↑	53	7	37	83.0
Gcr1	29.18	14.05	2.8E−06		↑	227	51	109	70.5
Crz1	25.84	21.27	0		↓	201	25	116	70.1
Gis1	24.81	32.82	0		↓	193	7	150	81.3
Mig1	23.01	30.76	0		↓	179	9	97	59.2
Rcs1	30.72	20.24	0		↓	239	17	51	28.5
Rlm1	25.45	28.01	0		↓	198	11	172	92.4
Sut1	23.39	24.93	0	↓	↓	182	10	66	41.8
Xbp1	35.60	18.07	0	↓	↓	277	47	103	54.2
Rpn4	91.00	12.14	9E−15		↓	708	222	396	87.3
Cat8	7.07	31.07	3.3E−14		↓	55	2	43	81.8
Adr1	17.35	17.29	4.1E−09		↓	135	8	94	75.6
Hms1	23.26	15.37	9.2E−08	↓	↓	181	24	103	70.2
Mga1	17.10	14.98	2.5E−05	↓	↓	133	24	79	77.4
Eds1	3.86	22.06	4.5E−05		↓	30	7	8	50.0
Gal3	2.83	25.00	4.6E−05		↓	22	2	8	45.5
Znf1	2.70	23.33	0.00019		↓	21	4	2	28.6

^#^User (%), ratio of the target number of a TF and the total number of [*SWI*^+^]-DEGs; CK (%), ratio between the number of targets found in [*SWI*^+^]-DEGs and the number of total targets of a TF from YEASTRACT. The TF/targets linkage is based on genetic data published.

In *swi1∆* cells, the transcription is modified for 12 TF genes who have overrepresented targets among the identified DEGs, including 4 upregulated TF genes (*MIG2, YPR015C*, *MIG3, RME1*) and 8 downregulated TF genes (*SUT1, HMS1, XBP1, YHP1, HAP4, TYE7, YOX1, ACE2*). *MIG2* and *YPR015C* are upregulated and *SUT1, HMS1* and *XBP1* are downregulated similarly in [*SWI*^+^] cells (Fig. 2a, Table S2). A total of 867 target genes was found in the *swi1∆* DEGs (298 up/569 down). The upregulation of the 4 TF genes may account for the increased transcription of 126 DEGs and the reduced transcription of another 196 DEGs in *swi1∆* cells when these TFs function as activators and inhibitors, respectively. Similarly, the decreased transcription of the 8 TF genes can be used to explain the downregulation of 323 DEGs and the upregulation of 140 DEGs in *swi1∆* cells when they act as activators and inhibitors, respectively. Taken together, the altered transcription of these 12 TF genes may be responsible for the increased transcription of 140 DEGs (47.0% out of the 298 upregulated DEG targets) and the reduced transcription of 398 DEGs (70.0% out of the 569 downregulated DEG targets) in *swi1∆* cells (Table S2).

Our analyses thus suggest that transcriptional alteration of a group of TF genes is an important contributor of the observed transcriptomes in [*SWI*^+^] and *swi1∆* cells.

### Aggregation of TFs and DEGs in [*SWI*^+^] cells

**Figure 3:**
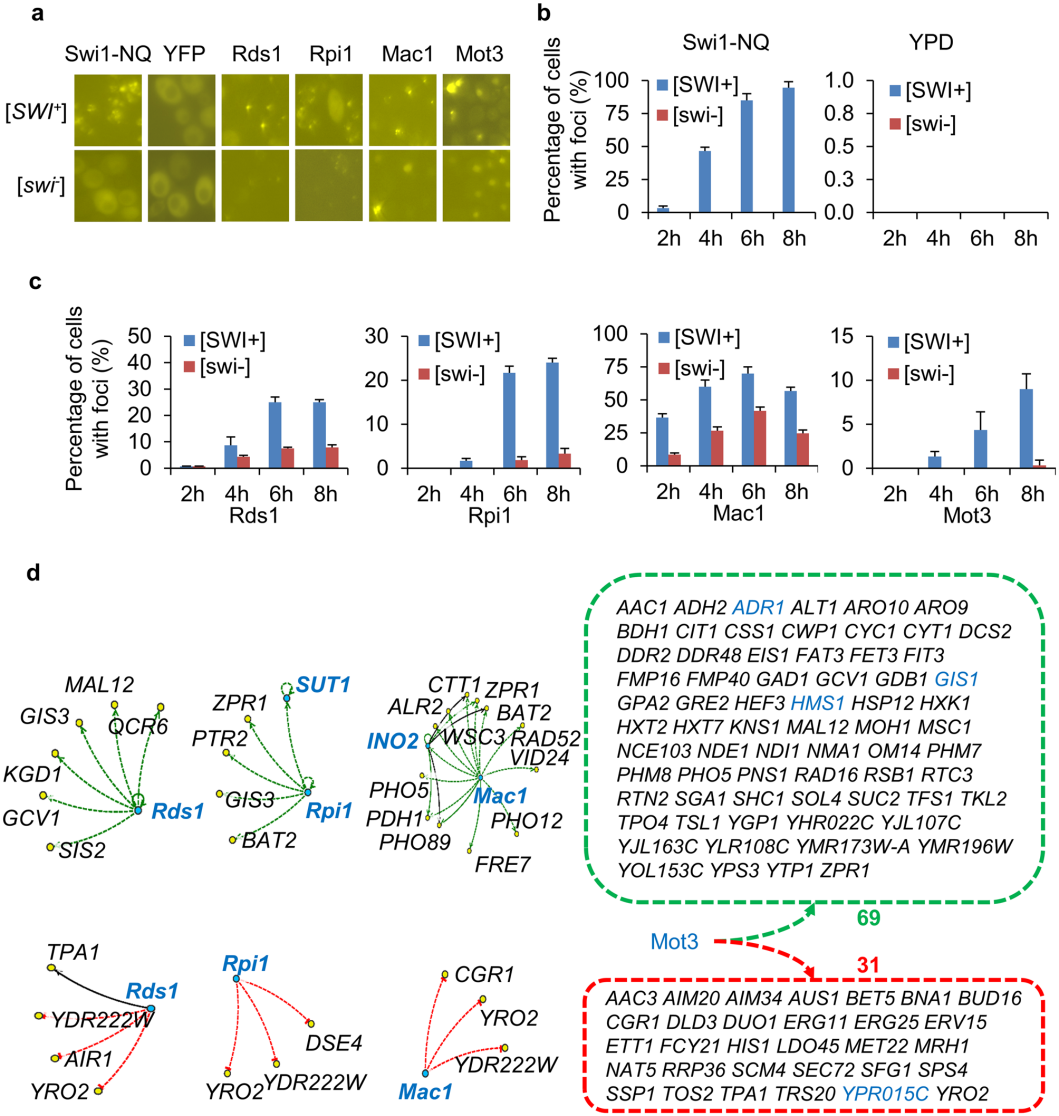
Contribution of higher aggregation propensities of 4 examined TFs to [*SWI*^+^] DEGs. (**a**–**c**) The 4 TFs are more aggregable in [*SWI*^+^] cells than in non-prion ([*swi*^*−*^]) cells among 30 tested TFs that were predicted having a higher aggregation propensity and with overrepresented targets in [*SWI*^+^]-DEGs (see Table S2 for details). To avoid unnecessary overproduction, 0.02% galactose was used to induce the expression of the indicated proteins from a single-copy plasmid. Representative images of the indicated YFP fusions are shown in panel (**a**), and quantitative results are shown in panel (**b**) (Swi1-NQ-YFP as positive control and YFP as negative control) and in (**c**) (for tested TFs). (**d**) Reduced activity of the 4 TFs due to aggregation explains the inhibition (upper) and activation (bottom) of [*SWI*^+^] DEG targets when they function as activators and inhibitors, respectively. All TFs in the regulatory network are highlighted by blue.

Next, we tested if conformational changes of TFs also contribute to the observed DEGs in [*SWI*^+^] cells. This test was inspired by our previous observation that besides Swi1, several additional transcriptional activators containing an asparagine-rich region(s), including Mss11, Sap30 and Msn1, are significantly aggregated in [*SWI*^+^] but not so in non-prion cells^31^. With focusing on TFs whose targets are significantly overrepresented within the [*SWI*^+^] DEGs, we examined their aggregation/prion-prone propensity. Utilizing two published algorithms, CamSol^49^ and PrionW^50^, which were designed for identifying aggregation-prone and prion-like proteins, 30 TFs with an intrinsic variant score < 1.5 using CamSol or a pWALTZ cut-off of > 73.5 and N + Q > 20% using PrionW were selected for further study (Table S2). Initially, isogenic [*SWI*^+^] and wt strains with individual TF genes endogenously tagged with GFP were used for the test. Unfortunately, we failed to see visible GFP foci for multiple TFs including Mss11, Sap30 and Msn1 that form amyloid- aggregates in [*SWI*^+^] cells^31^. We speculated that the endogenous level of these TFs is too low to visualize their aggregation. Thus, we turned to express their GFP fusions from single-copy plasmids driven by a galactose-inducible *GAL1* promoter. We observed that the aggregation of these fusion proteins indeed depended on their expression levels. With a galactose concentration of 2% (an overexpression condition), many of them formed foci in both [*SWI*^+^] and wt non-prion cells with varying frequencies (data not shown). After testing different galactose concentrations and induction times, 0.02% galactose and up to 8 h of induction were used in the formal experiments to avoid unnecessary overproduction. Under such conditions, Swi1-NQ-YFP (positive control) aggregated in [*SWI*^+^] but not in non-prion wt cells and YFP (negative control) did not aggregate in either type of cells (Fig. 3a,b). We observed dramatic increases in aggregation of four TFs—Mot3, Rds1, Mac1 and Rpi1 in [*SWI*^+^] cells while their aggregation frequency in wt (non-prion) cells was kept at minimal levels except for Mac1, which also significantly aggregated in non-prion cells but with a considerably lesser degree when compared to that in [*SWI*^+^] cells (Fig. 3a,c). The observed foci (aggregates) may reflect a change in protein localization, accumulation of amorphous aggregates, or formation of amyloids. Regardless of their nature, one consequence of such aggregations is the reduction of the functionality of these TFs and thus alterations of their target genes.

The four TFs are linked to 239 targets (168 downregulated/71 upregulated) among prion DEGs (Table S2). Nevertheless, only 86 (51.2% out of the 168 targets) downregulated genes and 34 (47.9% out the 71 targets) upregulated genes in [*SWI*^+^] cells are interpretable by the possible reduced activities of the four TFs due to aggregation (Fig. 3d and Table S2). Interestingly, Mot3, one of the four TFs, is the protein determinant of the [*MOT3*^+^] prion and is the most potent TF among the four TFs in regulating gene transcription, whose aggregation may account for 69 inhibited DEGs and 31 activated DEGs in [*SWI*^+^] cells (Table S2). The aggregation of Rpi1 explains the reduced transcription of 5 and the increased transcription of 3 target genes. The aggregation of Mac1 is perhaps responsible for the reduced transcription of 13 genes and the increased expression of 3 genes. Similarly, the aggregation of Rds1 might be the cause of the reduced transcription of 6 and, the increased expression of 3 genes (Fig. 3d and Table S2). Taken together, our data suggest that the aggregation of a group of TFs may be another contributory factor to our observed [*SWI*^+^] transcriptomic profile.

### Contribution of Swi1 interaction proteins to DEGs in [*SWI*^+^] cells

Swi1 interaction proteins are a group of proteins that work in concert with Swi1 physically or genetically to regulate the transcription of its target genes. We suspected that some Swi1 interaction proteins may be affected by [*SWI*^+^] and their functional alterations may also contribute to the observed DEG profile in [*SWI*^+^] cells. To examine if some of the Swi1 interaction proteins are subject to transcriptional or conformational changes in [*SWI*^+^] cells, we retrieved 313 such proteins from the *Saccharomyces* Genome Database (SGD), among which 261 genetically and 62 physically interact with Swi1, and 10 can interact with Swi1 both genetically and physically. Intriguingly, the transcription of 24 Swi1 interaction proteins are modified in [*SWI*^+^] cells, 9 (Prp43, Dur1,2, Aro1, Scm4, Vma21, Mrx12, Ydj1, Gim3, Alg5) are upregulated, and 15 (Mrk1, YGR201C, Sdp1, Msc1, Hor7, Rck1, Gdh3, Rpn4, Sok1, Ino2, Mtl1, Lsc2, Ptk2, Xdj1, Abz1) are downregulated. Importantly, such transcriptional alterations occur mostly in [*SWI*^+^] but not in *swi1∆* cells, with exceptions for *PRP43* whose expression increases in [*SWI*^+^] cells but reduces in *swi1∆* cells, and 6 other genes (*MRK1, YGR201C, SDP1, MSC1, HOR7, RCK1*) that are downregulated in both [*SWI*^+^] and *swi1∆* cells. Furthermore, we observed that 11 Swi1 interaction proteins are also TFs whose targets are overrepresented within the [*SWI*^+^]-DEGs, including Gcn4, Hap4, Yap1, Mss1, Msn2, Stp1, Pdr1, Rpn4, Stp2, Stb3 and Tup1. These results suggest that altered transcriptional regulation of certain Swi1 interaction protein genes may contribute to the DEGs in [*SWI*^+^] cells.

### Coordinating the effects of aggregation of Swi1 and TFs in [*SWI*^+^] cells

We reported previously that the Swi1 function is compromised in [*SWI*^+^] cells due to formation of nonfunctional prion aggregates^16,31^. After establishing that the [*SWI*^+^]-specific transcriptional profile is at least partially attributable to the aforementioned transcriptional and conformational alterations of related TFs and Swi1 interaction proteins, we next investigated how such alterations and a reduced Swi1 function in [*SWI*^+^] cells work together to define the [*SWI*^+^] transcriptome.

**Figure 4:**
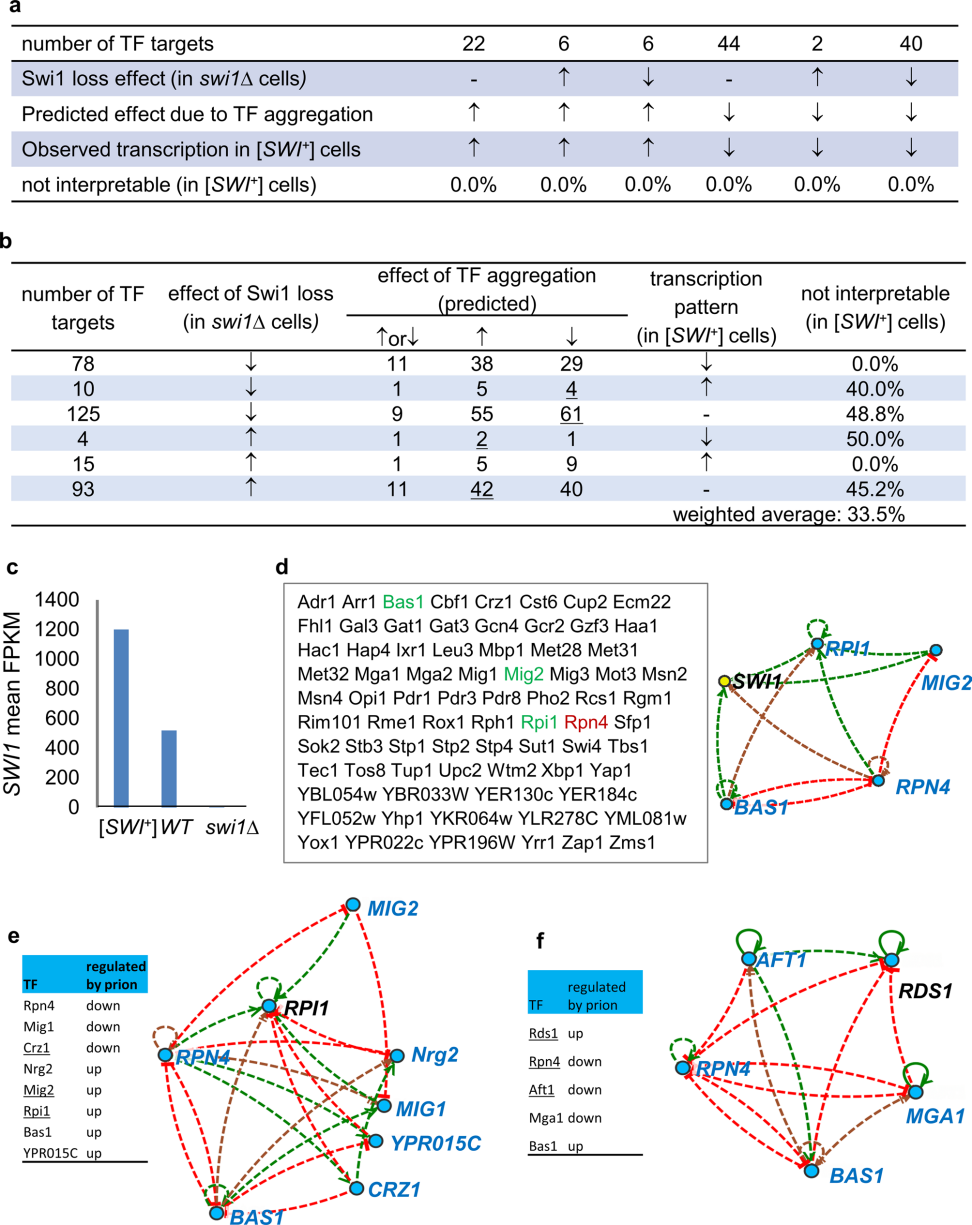
Coordinated aggregation effects of Swi1 and the 4 examined TFs on [*SWI*^+^]-DEGs. (**a**) The observed 120 [*SWI*^+^]-DEGs that are targets of the 4 aggregation-prone TFs are interpretable by a combined effect of Swi1 function reduction (predicted by their transcription in *swi1∆* cells) and aggregation of the 4 TFs (predicted by YEASTRACT). (**b**) Similarly, 325 *swi1∆*-regulated genes that are targets of the 4 aggregation-prone TFs were analyzed to justify the effects of aggregation (thus reduced function) of Swi1 and the 4 TFs on their transcription in [*SWI*^+^] cells. The transcription patterns of the *swi1∆*-regulated genes in [*SWI*^+^] cells are interpretable only for a fraction of these genes, and the number of genes not interpretable by aggregation of Swi1, TFs or a combination are underlined. (**c**) The transcription of *SWI1* gene is elevated in [*SWI*^+^] cells based on RNA-seq data. (**d**) Among the identified 76 *SWI1* regulators according to SGD, three are upregulated (green), and one is downregulated (red) in [*SWI*^+^] cells. Such regulations can be used to explain the elevated transcription of *SWI1* gene in [*SWI*^+^] cells. (**e**,**f**) The upregulation of *RPI1* and *RDS1* in [*SWI*^+^] cells, which encode two aggregation-prone TFs (Rpi1 and Rds1), can be explained by transcriptional alterations of their regulators, especially those underlined TFs. E, *RPI1* regulators, and F, *RDS1* regulators. In panels of (**a**,**b**), ↑, activation; ↓, inhibition; −, no transcriptional change. In panels of (**d**–**f**), dotted green lines denote activation, dotted red lines denote inhibition, and dotted brown lines denote activation / or inhibition based on expression evidence from literature, and all TF genes are highlighted in blue in the shown regulatory networks.

We first examined if the observed transcriptional changes of the 120 [*SWI*^+^] DEGs that are targets of the four aggregation-prone TFs shown in Fig. 3d can be interpreted by a combined effect of the aggregation of Swi1 and the four TFs. We reasoned that the regulatory effect of Swi1 aggregation on these DEGs in [*SWI*^+^] cells (with reduced Swi1 function) can be roughly estimated by their transcription in *swi1∆* cells (in the absence of Swi1). Meanwhile, the regulatory effect of TF aggregation (reduction in function) on these DEGs can be predicted using YEASTRACT. As shown in Fig. 4a, the 120 DEGs can be divided into 6 subgroups based on their expression patterns in *swi1∆* and [*SWI*^+^] cells. For genes that are similarly regulated in [*SWI*^+^] and *swi1∆* cells, such a similarity can be explained by the fact that the aggregation of the four TFs and the *SWI1* deletion have similar regulatory effects. For the other four subgroups of DEGs that are regulated differently by *swi1∆* and [*SWI*^+^], their transcriptional patterns can also be explained by a combined regulatory effect of the aggregation of Swi1 and the four TFs in [*SWI*^+^] cells (Fig. 4a).

In another analysis, we identified 325 *swi1∆-*regulated genes that are targets of the four TFs. Their transcription should be similarly modified in [*SWI*^+^] cells if the reduced Swi1 activity were the only regulatory factor. We found that 93 such DEGs show similar regulatory patterns (78 inhibited and 15 activated) in *swi1∆* and [*SWI*^+^] cells. For these genes, the effect of insufficient Swi1 function might be the primary cause. For the other four subgroups of genes (total 232), their transcription profiles are different in *swi1∆* and [*SWI*^+^] cells. As shown in Fig. 4b, the aggregation effects of Swi1 and the TFs are variable, and whether or not a regulatory pattern in [*SWI*^+^] cells is interpretable by the aggregation of Swi1 and the four TFs depends on individual genes. Overall, the transcriptional patterns may be explained by a combined effect of the compromised Swi1 function and/or the aggregation of TFs for 66.5% but not for the remaining 33.5% of DEG targets of the four TFs in [*SWI*^+^] cells (Fig. 4b).

Given that the expression of a substantial number of [*SWI*^+^] DEGs is not interpretable by the combined effects of Swi1 activity reduction and the TF aggregation, other alternative mechanisms may be involved. Interestingly, *SWI1* gene expression is upregulated ~ twofold in [*SWI*^+^] cells (Fig. 4c). There are 76 *SWI1* regulators in the genome based on data retrieved from SGD (Fig. 4d). Among them, the expression of Bas1, Mig2, and Rpi1, which are Swi1 activators, is upregulated, while Rpn4, a Swi1 repressor, is downregulated in [*SWI*^+^] cells (Fig. 4d left). These 4 altered TFs may form a regulatory network to result in *SWI1* upregulation in [*SWI*^+^] cells, and such an upregulation may partially alleviate and neutralize the effect of Swi1 aggregation and further alter the transcriptome of [*SWI*^+^] cells. In addition, we also observed that the transcription of genes encoding Rds1 and Rpi1 that are prone to aggregate in [*SWI*^+^] cells is increased (Table S2). This may be explained by an altered expression of several TFs (Fig. 4e). In this regard, three of 44 *RPI1* regulatory genes are downregulated and five are upregulated in [*SWI*^+^] cells (Fig. 4e left). The potential effectors can be Crz1 (downregulated repressor), Mig2, and Rpi1 self (upregulated activators) among these regulators. Similarly, among the 27 *RDS1* regulators, two are upregulated and three are downregulated in [*SWI*^+^] cells, and the potential regulators causing the upregulation can be Rpn4 (upregulated activator), Aft1, and Rds1 self (downregulated repressors) (Fig. 4f). The upregulation of Rds1 and Rpi1 may neutralize the effects of their aggregation to certain extent. These data provide one additional regulatory mechanism that may impact the [*SWI*^+^]-transcriptome.

### GO term analysis for [*SWI*^+^] and *swi1Δ* DEGs

To further investigate the functional effects brought out by the Swi1 prion and *SWI1* deletion, we next performed pairwise Gene Ontology (GO) term enrichment analyses for biological process, molecular function, and cellular component. Dramatic differences were observed between the two strains. Three pathways—external encapsulating structure, metabolism of pyridine-derived compounds, and oxidation–reduction processes are noticeably enriched in both [*SWI*^+^] and *swi1∆* DEGs. Other enriched processes are [*SWI*^+^] (11 processes) or *swi1∆* (9 processes)*-*specific. Surprisingly, most of the 9 enriched processes for *swi1∆* are relevant to the translation except for pyridoxine metabolic process. In contrast, pathways impacted by [*SWI*^+^] are diverse with the pathway of metabolism of carbohydrates and antibiotics being the most significant one. Other enriched processes involve in the metabolism of small molecules, glutamine family amino acids and dicarboxylic acid, response to inorganic substances and external stimuli and respiration. In agreement with the large number of DEGs comparing the [*SWI*^+^] and *swi1∆* strains, these observations indicate that the two strains impose dramatically different impacts on cellular pathways and [*SWI*^+^] mainly confers gain-of-function phenotypes.

**Figure 5:**
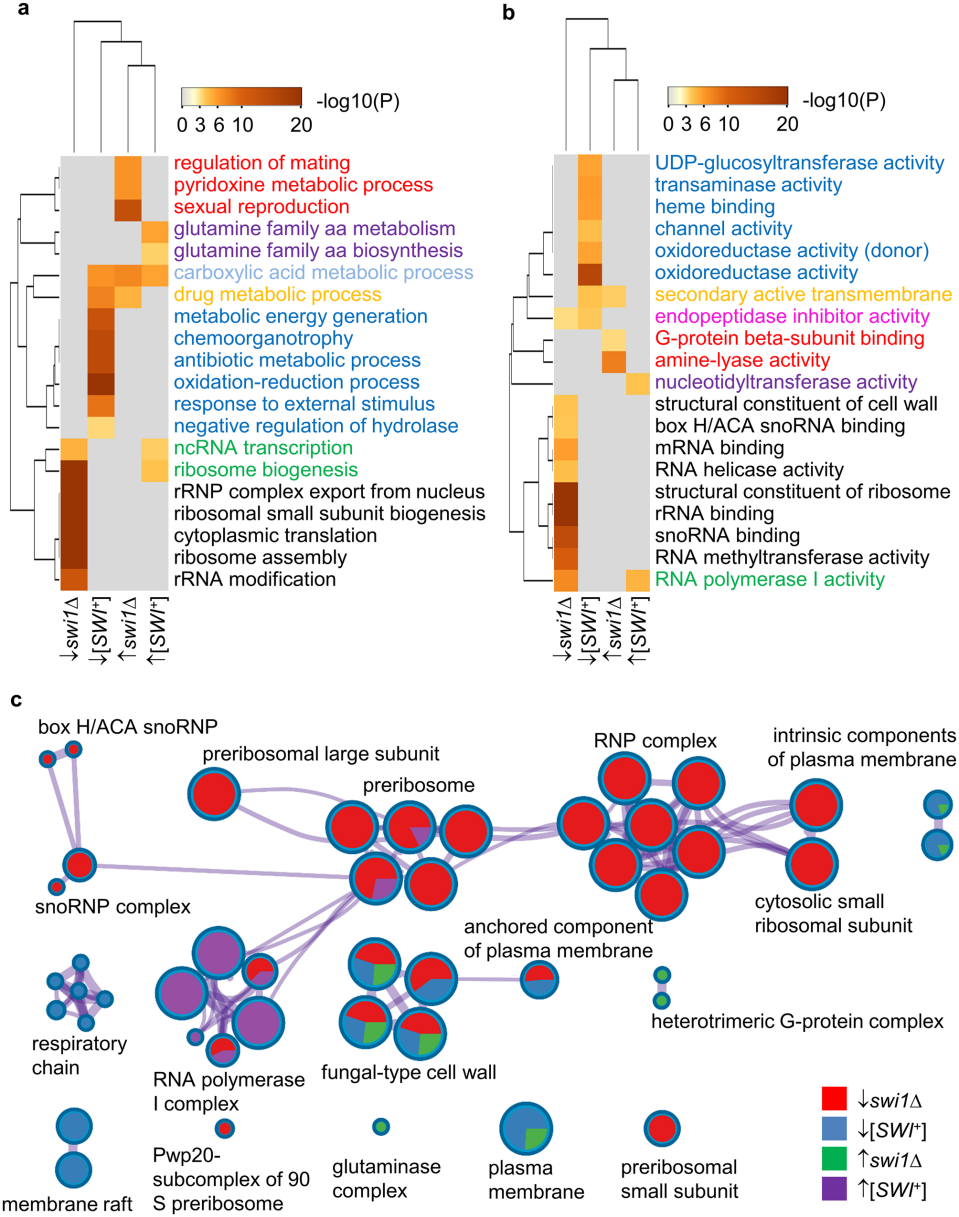
GO term enrichment analyses of [*SWI*^+^] DEGs and *swi1∆* DEGs. The enrichment of biological process (**a**) and molecular function (**b**) and cellular component (**c**) were analyzed for the four indicated subgroups of DEGs using Metascape (http://metascape.org). The cutoff for a significant enrichment is 0.001 as described in the Methods. In panels (**a**,**b**) the heatmaps are colored by their p-values as denoted, and the description of each term is highlighted by a specific color based on the enrichment pattern. For panel (**c**), enriched terms from the full cluster are converted into a network layout displayed by a circle node, where its size is proportional to the number of input genes fall into that term, and the color in each pie sector represent its regulatory patterns as shown. The thickness of an edge represents the similarity score. The network was visualized and edited with Cytoscape (v3.1.2).

Next, we performed similar enrichment analyses for four subgroups of DEGs—downregulated by [*SWI*^+^], upregulated by [*SWI*^+^], downregulated by *swi1∆,* and upregulated by [*SWI*^+^]. For the downregulated DEGs of [*SWI*^+^] and *swi1∆,* noticeable overlapping effects include enrichments in endopeptidase inhibitor activity, anchored plasma membrane components, and a subgroup of cell wall constituents (Fig. 5a–c).The enrichment for cell wall components is consistent with the lack of flocculin genes expression and our earlier reports on deficiency in multicellular features for both types of cells^31^. For the upregulated DEGs, both [*SWI*^+^] and *swi1∆* strains share an enrichment only in the carboxylic acid metabolic process. Other than the above common effects, most of the enrichments observed are specific to each of the four subgroups of our DEGs (Fig. 5). With regard to this notion, the most remarkable enrichment is in ribosome biosynthesis/assembly/transport, rRNA synthesis/processing and other translation-related events, which are specifically downregulated by *swi1∆* (Fig. 5). Interestingly, transcription of ribosome biogenesis genes is upregulated by [*SWI*^+^] (Fig. 5). These data suggest that the pathway of protein translation is largely impaired in *swi1∆* cells but may be promoted in prion cells. In contrast to the effects of *swi1∆,* the enriched pathways and functions for the downregulated DEGs in [*SWI*^+^] cells are diverse, and are mainly related to oxidation–reduction, respiratory chain and other energy generating metabolisms, and antibiotic metabolism and response to external stimulus. For DEGs upregulated by *swi1∆,* specific enrichments were noticed for pathways and functions relevant to sexual reproduction/mating, metabolism of pyridoxine, amino acids and G-protein signaling (Fig. 5). Moreover, for DEGs upregulated by [*SWI*^+^], specific enrichments were observed for RNA polymerase I-dependent ncRNA transcription and glutamine family amino acids biosynthesis (Fig. 5). When similar pair-wise comparisons of [*SWI*^+^]- and *swi1∆*-DEGs were expanded to a broader range of processes (top 100), similar conclusions can be drawn (Fig. S5). Our enrichment analysis results partially overlap with those reported by Malovichko et al*.* with substantial differences as will be discussed later^44^.

We next analyzed whether [*SWI*^+^] and *swi1∆* differentially regulate the pathway of carbohydrate utilization. There are four carbohydrate-related processes, including carbohydrate catabolic process, cellular carbohydrate metabolic process, oligosaccharide metabolic process, and carbohydrate transmembrane transport, which are all enriched in DEGs repressed in [*SWI*^+^] cells (Fig. 5 and S5). However, carbohydrate metabolic process is the only pathway merely enriched in genes upregulated in *swi1∆* cells. Thus, the carbon usage capacity may be impaired in [*SWI*^+^] but not in *swi1∆* cells. This seems to contradict our previous report that both *swi1∆* and [*SWI*^+^] cells are deficient in using non-glucose carbon sources^16^. To clarify this, further analyses were performed. We found that most identified DEGs that are involved in carbon usage are inhibited in both *swi1∆* and [*SWI*^+^] cells (Fig. 5 and Table S1). In [*SWI*^+^] cells, the inhibited genes encode proteins for maltose transporter (*MAL31*) and regulator (*MAL33*), hexose transporters (*HXT5, HXT2, HXT13, HXT6, HXT7*), glucose sensor (*SNF3*), regulators of non-fermentable sugar metabolism (*CAT8, GCY1, GUT1, YIG1, GUT2*), *GAL* gene activation (*GAL3, GAL4*), polysaccharide hydrolysis (*SUC2, MAL12*), glycolysis (*IDP2, PGM2, HXK1, GND2, SOL4, ZWF1*), tricarboxylic acid cycle (*CIT1, MDH1, PYC1*), glyoxylate cycle (*MLS1, ICL1, MDH2*), respiratory chain (*NDE2*), glycogen metabolism (*GIP2, GPH1, GLC3, IGD1, GDB1, SGA1, GLG1, GLG2*), glycogenesis (*GSY1, GSY2, PIG1, PIG2*), and trehalose metabolism (*NTH1, TPS1, TPS2, TSL1, ATH1*). Similarly, the inhibited genes in *swi1∆* cells include *SUC2, MAL31, MAL33*, *HXT4, HXT13, HXT6, HXT7,* and genes encoding proteins in glycerol synthesis (*GPD1*), glycolysis (*IDP2, PGM2, ENO2, TYE7*) and pentose phosphate pathway (*NQM1 RKI1*). Taken together, key genes in the carbohydrate metabolic pathways are inhibited in both *swi1∆* and [*SWI*^+^] cells to result in their deficiency in using non-glucose carbon source. Interestingly, *GAL1, GAL7* and *GAL10*, the three key genes required for catabolizing galactose are activated by *swi1∆* but not by [*SWI*^+^] (Table S1), suggesting a mechanistic difference in causing their deficiency in galactose usage in the two types of cells.

### The response of [*SWI*^+^] and *swi1∆* cells to stress

**Figure 6:**
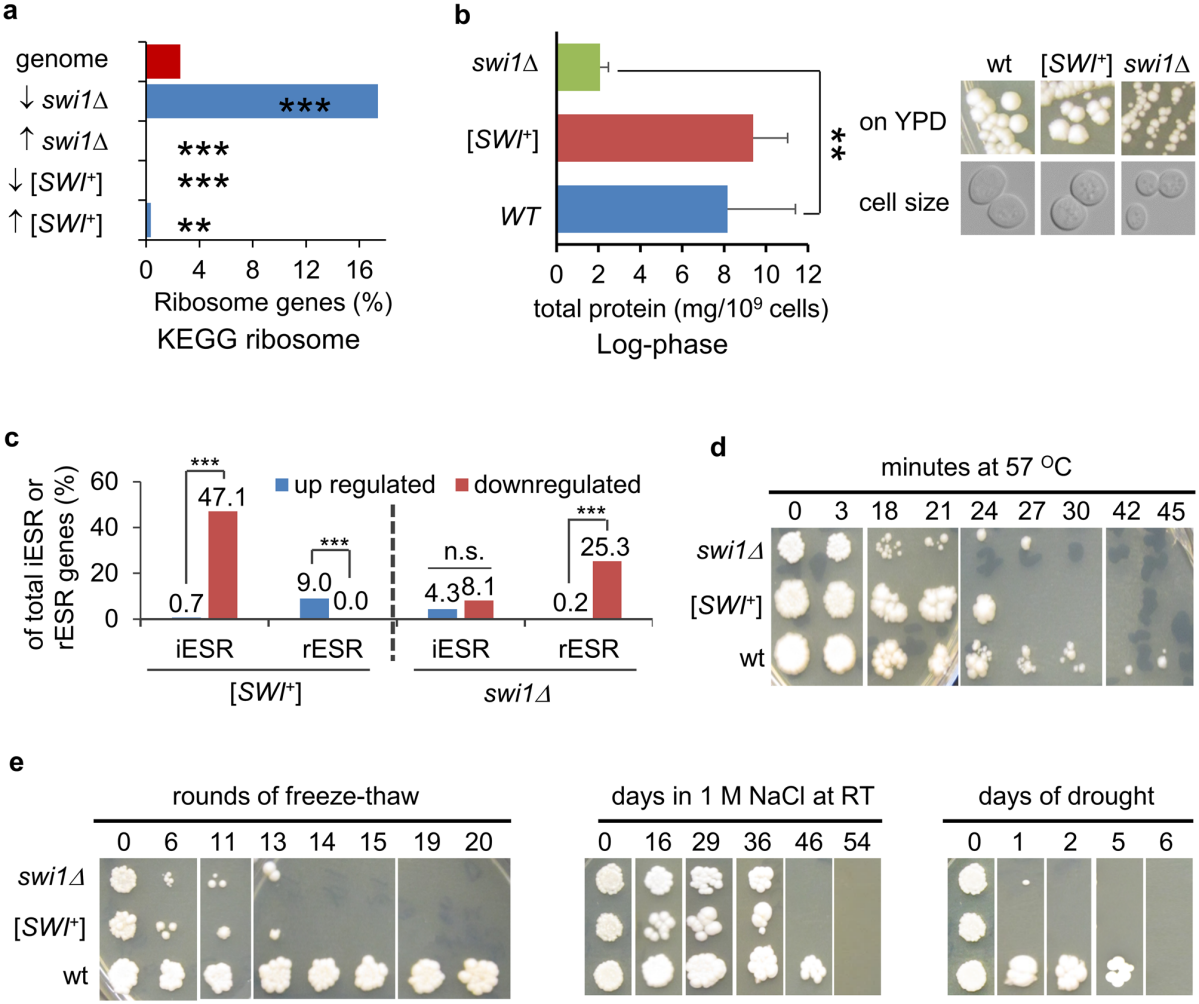
Analyses of protein synthesis and Environmental Stress Response (ESR) of *swi1∆* and [*SWI*^+^] cells. (**a**) KEGG ribosome gene enrichment analysis was performed as described in the Methods. ↑ and ↓ indicate upregulation and downregulation by *swi1∆* or [*SWI*^+^], respectively. (**b**) Left: shown are the steady state protein levels of the indicated strains. The total protein concentration normalized to cell number was determined for log-phase cells by a protein assay as described in the method. Right: images showing cell size and growth phenotype of the three strains used in this study. (**c**) Shown are fractions of environmental stress response (ESR) genes including iESR and rESR genes that are either activated or repressed in [*SWI*^+^] and *swi1∆* cells. The significance was analyzed by Fisher’s exact test for panels of (**a**,**c**) and Student T-test for panel (**b**). *p-value < 0.05; **p-value < 0.001; ***p-value < 0.001. (**d**,**e**) Shown are cell viability assays of the three tested strains upon the indicated treatments as described in the “Methods”.

Based on our GO term enrichment analysis predicting that ribosomes and translation-linked biological processes might be dysfunctional in *swi1∆* cells, we next carried out KEGG (Kyoto Encyclopedia of Genes and Gemomes) ribosome enrichment analysis for [*SWI*^+^] and *swi1∆* DEGs. As shown in Fig. 6a, an overrepresentation of KEGG ribosome genes was only seen for DEGs repressed by *swi1∆,* not for other three subgroups of DEGs. We speculated that *swi1∆* cells may produce reduced amounts of protein. To test that, steady-state levels of proteins from the three yeast strains were compared after normalizing to number of cells. As shown in Fig. 6b (left), although [*SWI*^+^] cells produced slightly more protein compared to wt, such a difference is not statistically significant. In comparison, *swi1∆* cells show significantly lower amount of total proteins. In agreement with this observation, we found that *swi1∆* cells indeed grow slower and have significantly a relative smaller cell size (Fig. 6b, right).

We next examined whether *swi1∆* and [*SWI*^+^] cells respond to environmental stresses differently. Under stressful conditions, yeast may activate defense systems, concurrent with slowing growth as a protection mechanism. Stresses may activate a common gene expression program called Environmental Stress Response (ESR)^51,52^, which is comprised of ∼ 300 induced (iESR) transcripts involved in stress defense and ∼ 600 repressed (rESR) transcripts important for cell division. Our analyses demonstrated that the iESR and rESR genes are differently regulated in *swi1∆* and [*SWI*^+^] cells (Fig. 6c). Specifically, a significantly larger number of rESR genes are activated (9.0%) rather than repressed (0.0%), while more iESR genes are significantly repressed (47.1%) rather than activated (0.7%) in [*SWI*^+^] cells. In other words, [*SWI*^+^] cells seem to have activated rESR genes but repressed iESR genes in YPD, a rich medium where the growth condition is supposed to be not stressful, implicating that [*SWI*^+^] cells may display a compromised ESR if cultivated in stressful conditions. Consistent with the characterization of slow growth, *swi1∆* cells significantly reduce the transcription of rESR genes (25.3% repressed and 0.2% activated) but do not significantly alter the transcription of iESR genes (8.1% repressed and 4.3% activated) (Fig. 6c). These data implicate that different from [*SWI*^+^] cells, *swi1∆* cells would have partially activated ESR even when they grow in a rich medium like YPD. These data suggest that [*SWI*^+^] and *swi1∆* cells may be mechanistically distinct in responding to stresses.

Based on the above analyses, we next performed experiments to test ESR of the *swi1∆,* [*SWI*^+^], and wt strains. Cells were subjected to transient thermal stress, high osmolality, or drought treatment. Cell survivability was then determined on a rich medium, YPD after a given period of recovery time. As shown in Fig. 6d,e, both *swi1∆* and [*SWI*^+^] cells showed greater sensitivities to these stresses than that of wt cells.

**Figure 7:**
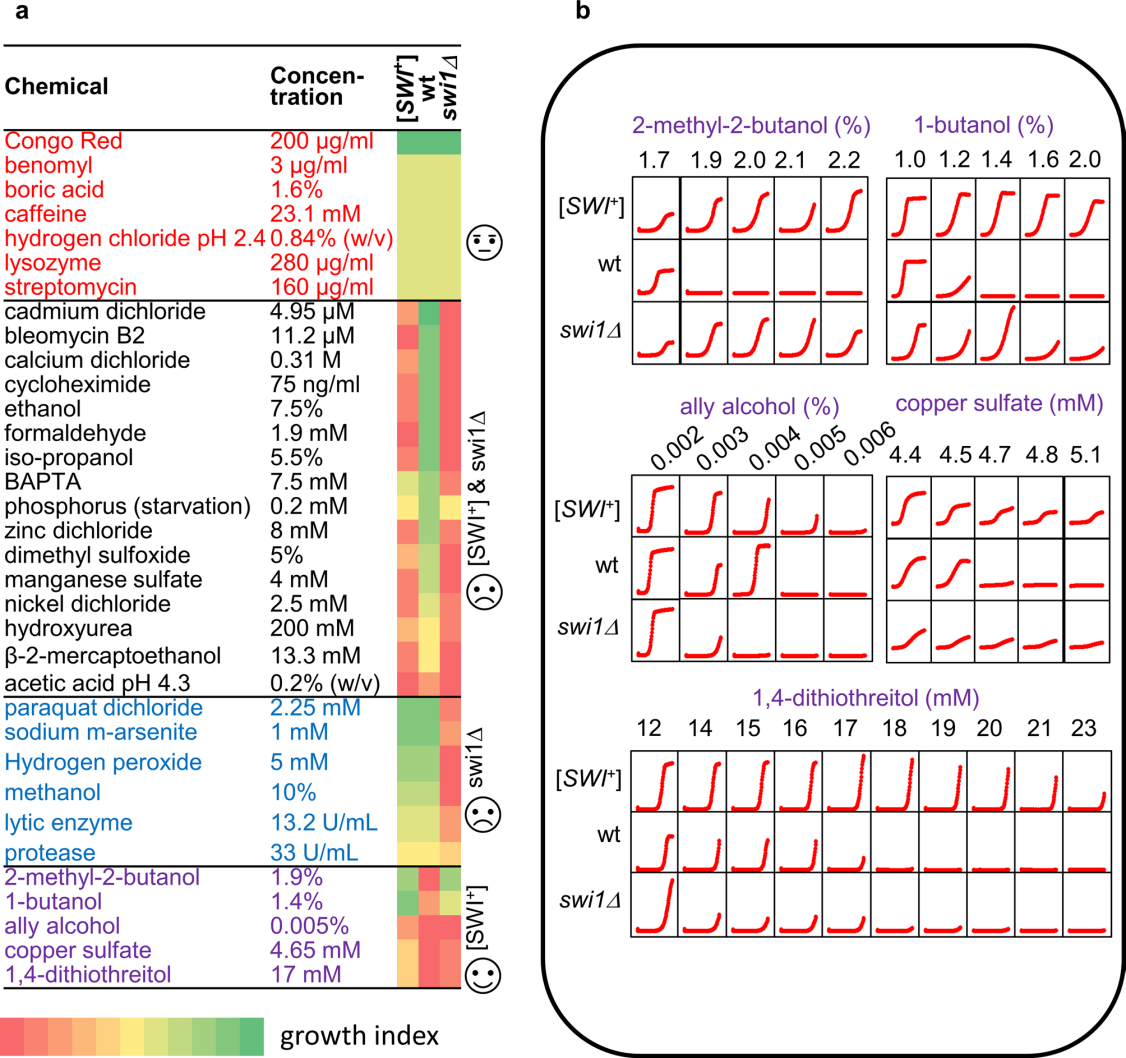
Growth analyses of the wt, [*SWI*^+^], and *swi1∆* strains upon treatment of a panel of chemical stressors. (**a**) Cell growth in YPD supplemented with various amount of each indicated chemical stressor was monitored by a plate reader in a time window of 72 h. The growth index (colored as denoted) was determined based on data from at least three independent tests as described in the Methods. The tested chemical stressors were categorized into 4 subgroups (indicated by different text colors) based on the response patterns of the three strains. (**b**) For chemical stressors that display a beneficial effect on the fitness of [*SWI*^+^] cells (purple-colored in the panel **a**), growth curves are shown for the three strains.

We then examined cells’ responses to various chemical stresses by growing them in liquid YPD medium supplemented with certain amounts of chemical stressors that were chosen based on literature from SGD (Fig. 7a). The fitness of cells was then determined by monitoring the growth curves (optical density at 600 nm—OD600) in a time window of 72 h. As shown in Fig. 7a, four growth patterns were discovered upon challenging with 34 stressors—no detectable differences, detrimental to both *swi1∆* and [*SWI*^+^], detrimental to *swi1∆* only, and beneficial to [*SWI*^+^] and/or *swi1∆*. We found that all three tested strains grew similarly for 7 chemical stressors (Congo Red, benomyl, boric acid, caffeine, hydrogen chloride pH 2.4, lysozyme, streptomycin), suggesting that no specific toxicities acted upon *swi1∆* and [*SWI*^+^] cells under these conditions. However, for 16 chemical molecules—cadmium dichloride, bleomycin B2, calcium dichloride, cycloheximide, ethanol, formaldehyde, iso-propanol, BAPTA, phosphorus, zinc dichloride, dimethyl sulfoxide, manganese sulfate, nickel dichloride, hydroxyurea, β-2-mercaptoethanol, acetic acid pH 4.3, most of these stressful conditions are detrimental to both *swi1∆* and [*SWI*^+^] cells in comparison to wild-type cells. Moreover, when challenged with 6 chemicals with the specified concentrations (paraquat dichloride, sodium m-arsenite, hydrogen peroxide, methanol, lytic enzyme, protease), a *swi1∆-*specific detriment was observed. Intriguingly, when supplemented with 5 additional chemical compounds, both *swi1∆* and [*SWI*^+^] cells grew better than wt cells though in some cases the difference is subtle (Fig. 7a bottom,b). Among the 5 compounds, 3 are alcohols—2-methyl-2-butanol, 1-butanol, and ally alcohol, while the other two are copper sulfate and 1, 4-dithiothreitol. Interestingly, ally alcohol is toxic to all three strains but [*SWI*^+^] cells showed a better tolerance. Taken together, our data suggest that the deletion and prionization of Swi1 can have a wide range of distinct phenotypic effects on cells under stressful conditions, which can be either neutral, detrimental, or beneficial.

## Discussion

The chromatin remodeling function by the ATP-dependent SWI/SNF complex is critical for various nuclear machineries to access DNA regions packed by histones and thereby essential for diverse cellular activities in the nucleus such as transcription, DNA replication, recombination and repair^22,53^. In this study, we have demonstrated that when Swi1, an important subunit of SWI/SNF, adopts a prion conformation to become [*SWI*^+^], it exhibits a distinct transcriptional profile compared to that of wt and *swi1∆* cells. Such a prion-specific transcriptomic signature is tightly associated with the altered transcription of genes encoding for TFs and Swi1 interacting proteins and the aggregation of numerous TFs. These prion-mediated modulations coordinate with substantial changes in various cellular pathways and functions. In contrast to a simple decline of protein translation-linked processes in *swi1∆* cells, functional alterations caused by [SWI^+^] are predicted to be largely distinctive to those caused by swi1∆. We show that [SWI^+^] and swi1∆ cells exhibit diverse and complicated stress responses, indicating that the two strains may adopt distinct mechanisms in ERS.

In this study, we identified that ~ 15.7% of yeast genes are differentially expressed in *swi1∆* cells (Fig. 1). Interestingly, an earlier study performed by Sudarsanam et al. only identified about 5% DEGs in *swi1∆* cells using a similar cutoff, and only 39.3% of DEGs from their study overlap with ours^54^. This discrepancy might be partially explained by the fact that the *swi1∆ *S288C strain used by Sudarsanam et al*.* may be a *FLO8* mutant while ours was repaired for this mutation. Perhaps, the major difference between the two studies is that Sudarsanam et al. used DNA microarray while we used RNA-seq.

A very recent study performed by Malovichko et al*.* has also compared the transcriptomes of strains of wild-type, [*SWI*^+^], and *swi1∆* using RNA-seq^44^. From their study, 27.5% DEGs were identified from *swi1∆* cells^44^, significantly more than ours; and only 25.2% of them overlap with ours. Surprisingly, only 59 DEGs were uncovered from [*SWI*^+^] cells in their study, and only 34.4% of them overlap with ours despite the fact that a similar DEG calling criterium was used^44^. In contrast to their observation of a reduced expression of *SUP45* in [*SWI*^+^] and an elevated expression of *ade1-14* in *swi1∆* cells^44,55^, we found that *SUP45* transcription is not altered and that *ADE1* expression is rather mildly decreased in *swi1∆* cells. With regard to *PER33, ICS2, DLD3*, and *ENA1* that were shown to be uniquely upregulated in [*SWI*^+^] cells in their study^44^, we found that the transcription is not altered in [*SWI*^+^] cells for *PER33,* but reduced only in *swi1∆* cells for *ICS2,* and increased for *DLD3* or reduced for *ENA1* in both *swi1∆* and [*SWI*^+^] cells (Table S1). For the three genes, *FMP16, AGX1*, and *IDP2,* reported by Malovichko et al*.* that are uniquely downregulated in [*SWI*^+^] cells, we confirmed that it is the case for *FMP16* and *AGX1* but *IDP2* is downregulated in both *swi1∆* and [*SWI*^+^] cells. As to the two genes*, HXY5* and *HSP12,* shown to be upregulated by [*SWI*^+^] but downregulated by *swi1∆*^44^, we found that it is true for *HXY5*; however, *HSP12* is downregulated similarly by both *swi1∆* and [*SWI*^+^] (Table S1). Regarding this, chromosome I disomy may be a contributory factor for their observed change in *HSP12* and *HXT5* transcription in *swi1∆* cells since it can lead to upregulation of *OAF1* that encodes a TF activating the two genes^44^. In contrast to the Malovichko et al*.* study, we identified a large number of genes that are uniquely regulated either in *swi1∆* or [*SWI*^+^] cells – 15 genes activated by [*SWI*^+^] but inhibited by *swi1∆*, and 32 genes inhibited by [*SWI*^+^] but activated by *swi1∆* (Fig. 1e). As to GO term enrichment analyses (Fig. 5 and^44^), one common finding between ours and the Malovichko et al*.* study is that most of the enriched processes and functions are specific to each of the four subgroups of DEGs from [*SWI*^+^] *swi1∆* cells. Both studies predicted that translation-associated pathways are repressed while G-protein signaling may be promoted by *swi1∆,* and a negative regulation of both [*SWI*^+^] and *swi1∆* on carbohydrate metabolism. Nevertheless, substantially different results were obtained in the two studies. For example, we observed that pathways of cell wall synthesis were downregulated by both [*SWI*^+^] and *swi1∆,* which were not seen by Malovichko et al*.* Several downregulated processes by both [*SWI*^+^] and *swi1∆* were shown in Malovichko et al*.* were not noticed in our study. In addition, the upregulated pathways by both [*SWI*^+^] and *swi1∆* in this study were not found by Malovichko et al. Moreover, for DEGs upregulated by [*SWI*^+^], we found a few enrichments while Malovichko et al. did not observe any (Fig. 5 and^44^). These discrepancies can be at least partially explained by different strains and cultivating conditions being used; Malovichko et al*.* used strains that may carry a mutated *FLO8* gene and a functionally altered *SUP*35 gene^44^. Our strains carry no alterations at the *SUP35* locus. In addition, chromosome I disomy of the *swi1∆* strain in their study was not observed for our strains. Further, yeast cells were cultivated in a minimal medium containing galactose as sole carbon source in which both the *swi1∆* and [*SWI*^+^] strains would grow poorly in their study while our strains were grown in YPD, a standard rich medium. Therefore, the impact of different Swi1 states is condition- and genetic background-specific.

As a global gene regulator, the SWI/SNF complex directly occupies promoter regions of ∼ 10% of yeast genes^24^, however, we identified a much larger number of DEGs from [*SWI*^+^] and *swi1∆* cells (Fig. 1a). In addition, the transcriptomic effects of [*SWI*^+^] and *swi1∆* can be distinct from different studies as discussed earlier. We believe secondary responses under specific cellular and environmental conditions besides the functional reduction of Swi1 may have specified such distinctions. For example, we found that the transcriptional and conformational alterations of TFs and Swi1 interacting proteins are important contributors of the modified transcriptome of [*SWI*^+^] cells. Interestingly, we also found that the transcription of Swi1, Rds1, and Rpi1, which are aggregation-prone in prion cells, is promoted (Fig. 4), suggesting a feedback regulation mediated by TFs. Since Swi1 activity reduction, aggregation of the TFs, and their combination cannot fully interpret the transcriptional alterations of their targets, we suspect that other alternative mechanisms may be involved, such as modifications on the integrity, functionality or specificity of the SWI/SNF complex and aggregation of other unknown proteins in [*SWI*^+^] cells.

A recent study shows that there are 32 TF genes that are differently regulated in several mutants of SWI/SNF subunits (19 inhibited and 13 activated), and two of them, *MET28* and *MET32* contribute to Snf2 occupancy and transcriptional alterations following the loss of Snf5 and Swi3^26^. Unfortunately, the *SWI1* deletion strain was not included in that study. Nevertheless, we have noticed that none of the 4 activated TF genes in *swi1∆* cells from this study belong to the 32 TF genes, whereas among the 8 *swi1∆*-inhibited TF genes (Fig. S2), *HMS1*, *TYE7,* and *HAP4* are similarly inhibited in those SWI/SNF subunit mutants ^26^. Among the 7 [*SWI*^+^]-activated TF genes, *NRG2* and *YPR015C* are also activated in some of the mutants, however, *RPI1* is inhibited and others are not changed in those mutants. *NRG2* and *RPI1* are also activated by *swi1∆* in this study, suggesting that they are common inhibitory targets of these SWI/SNF subunits. Among the 15 [*SWI*^+^]-inhibited TF genes, *HMS1* is also inhibited by *swi1∆* and by a few mutants of other SWI/SNF subunits, suggesting that an intact SWI/SNF complex is essential for its activation; however, instead of being inhibited, *CAT3, MGA1, EDS1,* and *GAL3* are activated in those SWI/SNF mutants. These 4 TF genes may have specific contributions to the altered transcriptome of [*SWI*^+^] cells. It was proposed that loss of some individual SWI/SNF subunits does not necessarily cause the collapse of the complex; instead, it can result in formation of altered sub-complexes that may significantly modulate Snf2 occupancy and thus alters the transcriptomic profile (e.g., number and regulatory pattern of DEGs)^24–26,56^. However, the effect of Swi1 loss on SWI/SNF architecture and Snf2 occupancy has not yet been tested. The lack of related data makes it less convincing to designate Swi1 as a separate module of the complex. Swi1 can interact with TFs individually^57^ and can be targeted to the *ARG1* promoter in the absence of Snf2^58^. In HeLa S3 cells, human Snf5 and Swi3 can be targeted to distinct DNA loci that are not bound by Snf2^59^. Under hypoxia, Snf11 and members of the Snf5/Swi3 regulatory module can migrate to the cytoplasm, whereas others may remain in the nucleus^60^. Thus, it is possible that Swi1 may act by itself or collaborate with some of the members of the SWI/SNF complex in regulating the transcription of genes in alternative loci. In [*SWI*^+^] cells, the aggregation of TFs in addition to Swi1 prion aggregation adds an extra layer of regulatory complexity. In addition to the three TFs we reported previously^31^, Mss11, Msn1, and Sap30, we have identified four additional TFs that are aggregated in [*SWI*^+^] cells (Fig. 3). Of the TFs, Mot3 is particularly interesting as it is the protein determinant of the prion [*MOT3*^+^]^14^. Our result implicates that Swi1 and Mot3 may interact during the prionogenesis process and that they are likely to influence each other’s de novo appearance and propagation. It remains unclear to what extent the SWI/SNF complex recruits the monomeric or oligomeric forms of Swi1 and how the structure and activity of the complex are altered in the context of [*SWI*^+^]. It would be interesting to explore in future studies if chromatin interactions and histone marks shown as important factors of chromosome remodeling and gene expression^61^, are also modulated in [*SWI*^+^] cells.

One of the most dramatic findings in this study is that genes for RNA processing, ribosome biogenesis and assembly, and other translation-related processes are severely inhibited in *swi1∆* cells (Figs. 5, 6 and Table S1). Earlier studies showed that the loss of Snf5, Swi3, or Swi1 also has similar defects^26,44,62^. These data suggest that SWI/SNF is an important regulator of protein translation. Interestingly, most rESR genes are linked to translation, and stressed cells are programed to reduce cell division by inhibiting general protein production and to induce transcription of genes that are associated with cellular defense (mainly iESR genes)^52,63^. Our finding that *swi1∆* cells inhibit rESR genes (Fig. 6c) suggests that *swi1∆* cells may be partially under stress even in rich medium (YPD). Interestingly, [*SWI*^+^] cells do not exhibit the defects in translation-related processes (Figs. 5, 6 and Table S1). On the contrary, ribosomal and rESR genes are activated in [*SWI*^+^] cells, suggesting that cell growth is promoted in [*SWI*^+^] cells. The fact that iESR genes are inhibited in [*SWI*^+^] cells suggests the stress response is not triggered but rather somehow repressed in the prion cells. Taken together, *swi1∆* and [*SWI*^+^] cells may mechanistically adopt different strategies in ESR even though the outputs of ESR can be similar for the two strains (Figs. 6, 7). In addition, genes repressed by [*SWI*^+^] have an overrepresentation of genes associated with the positive regulation of ion transport, response to water deprivation and salt stresses (data not shown), which may explain the sensitivity of [*SWI*^+^] cells to thermal and osmotic stresses, and drought treatment (Fig. 6). This is consistent with earlier observations that Snf2, Snf5 and Taf14 are involved in the stress alleviation^64,65^. Other than this, diverse responses of *swi1∆* and [*SWI*^+^] cells to a variety of chemical stresses highlights the complexity of ESR mediated by the Swi1 prion and its null mutant.

Taken together, our study has revealed substantially differences of the transcriptomic profiles of [*SWI*^+^] and *swi1∆* cells, and important mechanisms contributing to the acquired transcriptional fingerprint and gain-of-function phenotypes of [*SWI*^+^] cells. Our results may not only provide insight into mechanism governing the prion-mediated transcriptional regulation in yeast but also offer mechanistic clues for deciphering the pathogenesis of other aggregation-associated human diseases.

## Methods

### Strains media, oligo primers and plasmids

Wild-type non-prion ([*swi*^-^] or wt), [*SWI*^+^] and *swi1∆* strains used in this study were created and reported previously^31^. These isogenic *Saccharomyces cerevisiae* strains (*MATa his3Δ1 leu2Δ0 met15Δ0 ura3Δ0 flo8 FLO8-HIS3*) are all S288C derivatives that are *FLO8-*repaired to enable the flocculin (*FLO*) gene expression and the only difference among them lies in the Swi1 state: *swi1∆*—the chromosomal *SWI1* is deleted; [*SWI*^+^]—wt genome but Swi1 is in prion conformation; and wt—wt genome and Swi1 is in non-prion conformation.

Standard yeast-cultivating rich media (YPD) or synthetic complete (SC) media with defined amino acid supply for selection were used in this study. For raffinose phenotype assay, 2% raffinose was the only carbon source in the SC media, which was supplemented with 0.5 µg/ml antimycin (Sigma-Aldrich, St. Louis, MO). Yeast strains were grown at 30 °C unless otherwise defined. Bacterial strains were grown in LB medium at 37 °C.

Plasmids in this study include *p416TEF-NQYFP*^27^*, p416SWI-SWI1*^16^*, p416TEF* (ATCC), *pRS316CUP1*^43^* and pCUP1-RNQ1GFP*^43^*.* Thirty TF genes and YFP were PCR amplified and cloned into *SpeI/XmaI, XbaI/XmaI* or *NheI/XmaI* sites of *p415GAL1-NQYFP*^31^, resulting in YFP-fusion plasmids for their *GAL1* promoter-controlled expression (see Table S2 for primer list). All plasmids were verified by restriction cleavage and DNA sequencing.

### RNA-seq, read processing, and differentially expressed genes (DEG) calling

Overnight cultures in YPD were diluted to ~ 10^5^ cells/mL with fresh YPD. After 6 h of growth, cell concentrations of log-phase cultures were quantified and harvested. Total RNA was then extracted with RNeasy Mini Kit (Cat. No. 74104, QIAGEN) using protocols provided by the manufacturer. To avoid unnecessary perturbation of cellular metabolism that may alter gene expression profiles during enzymatic lysis, cells were lysed by bead-beating not by the recommended enzymatic lysis as described in the manufacturer protocol. Lysates were treated twice with DNase I to ensure a complete removal of genomic DNA. RNA QC and quantitation was performed using the Agilent Bio-analyzer 2100.

RNA-seq was conducted at University of Chicago Genomics Facility (http://genomics.bsd.uchicago.edu/) with three bio-repeats for each of the three strains. In brief, libraries were generated using the Illumina TruSEQ mRNA kit as protocolled by Illumina. Libraries were quantitated using the Agilent Bio-analyzer and sequenced on an Illumina HiSEQ2500 as protocolled by Illumina with a reading type of bidirectional 50-bp.

RNA-seq reads were trimmed to remove low quality trailing sequence using Trimmomatic 0.33^66^. Reads were then aligned to the sacCer3 assembly of the *Saccharomyces cerevisiae* genome with Tophat 2.1.0^67^ and attributed to genes using HTSeq 0.6.1^68^. The depth of the sequencing was roughly estimated from the length of *S. cerevisiae* genome (G), the number of reads (N), and the average read length (L) as NxL/G. Differentially expressed genes were called using EdgeR^69^, with an adjusted p-value cutoff < 0.001 and |log2(FC)|> 1. We used the plotMDS function from EdgeR to assess the variance between samples, and two duplicates for each strain were used for final DEG calling.

### Computational analyses

Comprehensive integrated biological information for the budding yeast *Saccharomyces cerevisiae* S288C was retrieved from the *Saccharomyces* Genome Database (SGD). The Venn diagram was generated using an online tool (http://bioinformatics.psb.ugent.be/webtools/Venn/). YeastMine^70^ was used to generate gene lists and analyze the distribution and features of genes of interest lists. TF enrichment was performed using YEASTRACT^71,72^ with a cut-off of p-value smaller than 0.001 based on DNA-binding plus expression evidence. The TF/target associations and regulatory networks were visualized with a force-directed layout. Aggregation-prone TFs were predicted with CamSol^49^ and PrionW^50^, and 30 TFs were selected for aggregation assay when the intrinsic variant score is smaller than 1.5 with CamSol, or the pWALTZ score is larger than 73.5 and an N + Q content higher than 20% with PrionW. Metascape^73^ was used for the Gene Ontology (GO) enrichment analysis. The enrichment cut-off used in this study was set to > 3 for Min Overlap, < 0.001 for p-value and > 1.5 for Min Enrichment. Gene Prioritization by Evidence Counting (GPEC, beta testing) was performed, otherwise a few analyses were also performed with a p-value of < 0.01. The visualization of enriched GO pathways was edited and optimized with Cytoscape 3.7.1^74^. To test if a gene list contains overrepresented TF genes, the ratio of TF genes in a user list was compared to the ratio of total TF genes (183 total TF genes defined by YEASTRACT) in the yeast genome (7185 genes), and Fisher’s exact test was used to calculate the significance. Similarly, the overrepresentation of environmental stress response (ESR) genes including ~ 300 induced (rESR) and ~ 600 reduced (rESR) transcripts^51^ as well as KEGG ribosome genes (https://www.genome.jp) in user gene lists were analyzed, and the significance was calculated with Fisher’s exact test. In these significance tests, the cut-off of the p-value is < 0.05.

### Growth assays

Since *swi1∆* and [*SWI*^+^] cells exhibit abolished or reduced ability in using non-glucose carbon sources such as raffinose, glycerol and galactose^16^, the growth of *swi1∆* and [*SWI*^+^] strains were assayed on SC agar plates containing the indicated carbon source. After spreading cells onto plates with the indicated carbon source, images were taken after 3 days of incubation at 30 °C. As control, the *swi1∆* strain was also transformed with *p416SWI-SWI1,* a single-copy plasmid expressing *SWI1* from its endogenous promoter or an empty vector to verify the phenotypic complementation in similar growth assays using SC media without uracil. The effect on cell growth upon heat shock, freeze–thaw, drought, or high osmolarity stress was analyzed on semisolid agar plates. For heat shock treatment, properly diluted log-phase YPD cultures of *wt, swi1∆* and [*SWI*^+^] strains were incubated at 57 °C and spotted onto YPD plates at different time points. For freeze–thaw treatment, the same YPD cultures, part of which was used for heat-shock treatment, were frozen at − 80 °C and thawed at 42 °C for number of cycles as indicated before spotting onto YPD plates. In the drought assay, cells from the same cultures were pelleted by spinning down at 2500 rpm for 3 min, kept at room temperature for different days after removing the supernatant, and were spotted onto YPD. To examine cell viability in responding to osmotic stress, cells derived from log-phase YPD cultures were properly diluted into YPD liquid medium supplemented with 1 M NaCl, incubated at room temperature for a different number of days before spotting onto YPD plates. In these assays, the cell survivability was estimated after 3 days of growth. To examine cell growth in response to chemical stressors, overnight YPD cultures of wt, *swi1∆*, and [*SWI*^+^] strains were counted and properly diluted into YPD liquid medium containing various amount of a chemical compound that is potentially toxic to yeast based on literature. The cell growth dynamics was then recorded by measuring the optical density at 600 nm (OD600nm) in a time course of 72 h.

### Aggregation assay

The aggregation states of each examined protein in *wt, swi1∆*, and [*SWI*^+^] strains were investigated by fluorescence microscopy^27,43^. In brief, the Swi1 state was assessed by examining the aggregation of Swi1-NQ-YFP expressed from a single-copy plasmid *p416TEF-NQYFP* that was controlled by the constitutive *TEF1* promoter*.* The Rnq1 ([*RNQ*^+^] determinant) status was verified by examining the aggregation status of Rnq1-GFP whose expression was governed by *CUP1* promoter from a single-copy plasmid *pCUP1-RNQ1GFP.* For Swi1, cells from SC-ura were directly observed while the Rnq1 aggregation was examined after growing cells in SC-ura medium containing 25 µM CuSO_4_. For both prion proteins, an empty plasmid was included as a negative control. To examine the aggregation propensity of 30 TF-YFP proteins that were under control of *GAL1* promoter from a single-copy plasmid, the *wt* and [*SWI*^+^] strains were transformed, and the log-phase cultures (in SC-leu containing 2% sucrose) of their transformants were induced with different amounts of galactose (from 0.01 to 2%) with different induction times. Induction with 0.02% galactose for up to eight hours was finalized in the formal test. The aggregation frequencies were then observed with a fluorescence microscope and quantified.

### Adhesive growth assay

Yeast multicellular phenotypes conferred by flocculin genes including adhesive growth on agar plates were confirmed based on methods described previously^31,75^. In brief, the tested strains were simply grown on YPD or SC selective plates, incubated for 3 days at 30 °C and then continued to incubate at room temperature for 3 days. Cells were then washed with water for different times with or without rubbing. Images were taken before and after washing.

### Protein quantification in cell lysates.

This was done based on a published protocol^76^. Briefly, overnight cultures of *wt, swi1∆* and [*SWI*^+^] cells in liquid YPD were properly diluted into the same medium and harvested at log and stationary phases. Cell pellets were collected after centrifugation at 660 g for 3 min and washed twice with water. Cell densities were determined after cell counting. Cells were disrupted by glass beads for 6 × 1 min using a bead-beater in lysis buffer (50 mM sodium phosphate buffer, pH 7.5, 100 mM NaCl, 1 mM dithiothreitol, 2 mM PMSF, 2 μg/mL pepstatin A, 2 μg/mL leupeptin, 5 μg/mL aprotinin and 1 mM benzamidine-HCl). Lysates were centrifuged at 500 g for 5 min at 4 °C to remove cell debris. Supernatants were transferred to new tubes and the protein concentration was determined using Pierce 660 protein assay reagent (Thermo Scientific, ProD# 22,660) based on a protocol provided by the manufacturer. Different concentrations of BSA were used as control to set the standard of protein concentrations. Multiple tests were performed with at least three bio-repeats in each test. Protein concentrations were then normalized to cell numbers as outcomes.

## Supplementary information

Supplementary Information 1.

Supplementary Information 2.

Supplementary Information 3.
