# Supplementary material for: Elucidating the regulatory mechanism of Swi1 prion in global transcription and stress responses
Source: Sci Rep. 2020 Dec 14;10:21838. doi: 10.1038/s41598-020-77993-0 (PMC7736884; doi:10.1038/s41598-020-77993-0)
Supplement: Supplementary file 1 — Supplementary Information 1. [file 41598_2020_77993_MOESM1_ESM.pptx]

## Slide 1
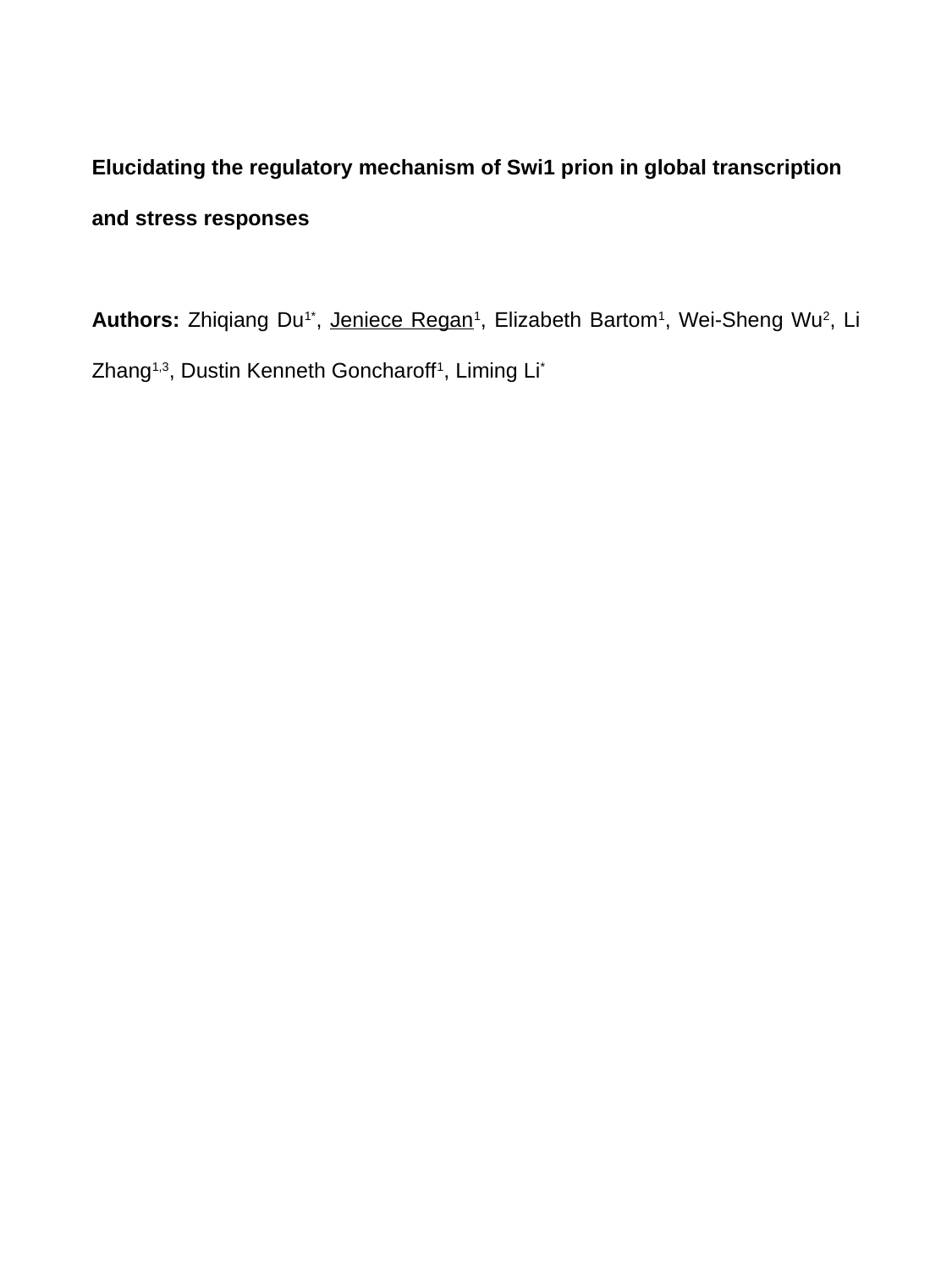

Elucidating the regulatory mechanism of Swi1 prion in global transcription and stress responses
Authors: Zhiqiang Du1*, Jeniece Regan1, Elizabeth Bartom1, Wei-Sheng Wu2, Li Zhang1,3, Dustin Kenneth Goncharoff1, Liming Li*

## Slide 2
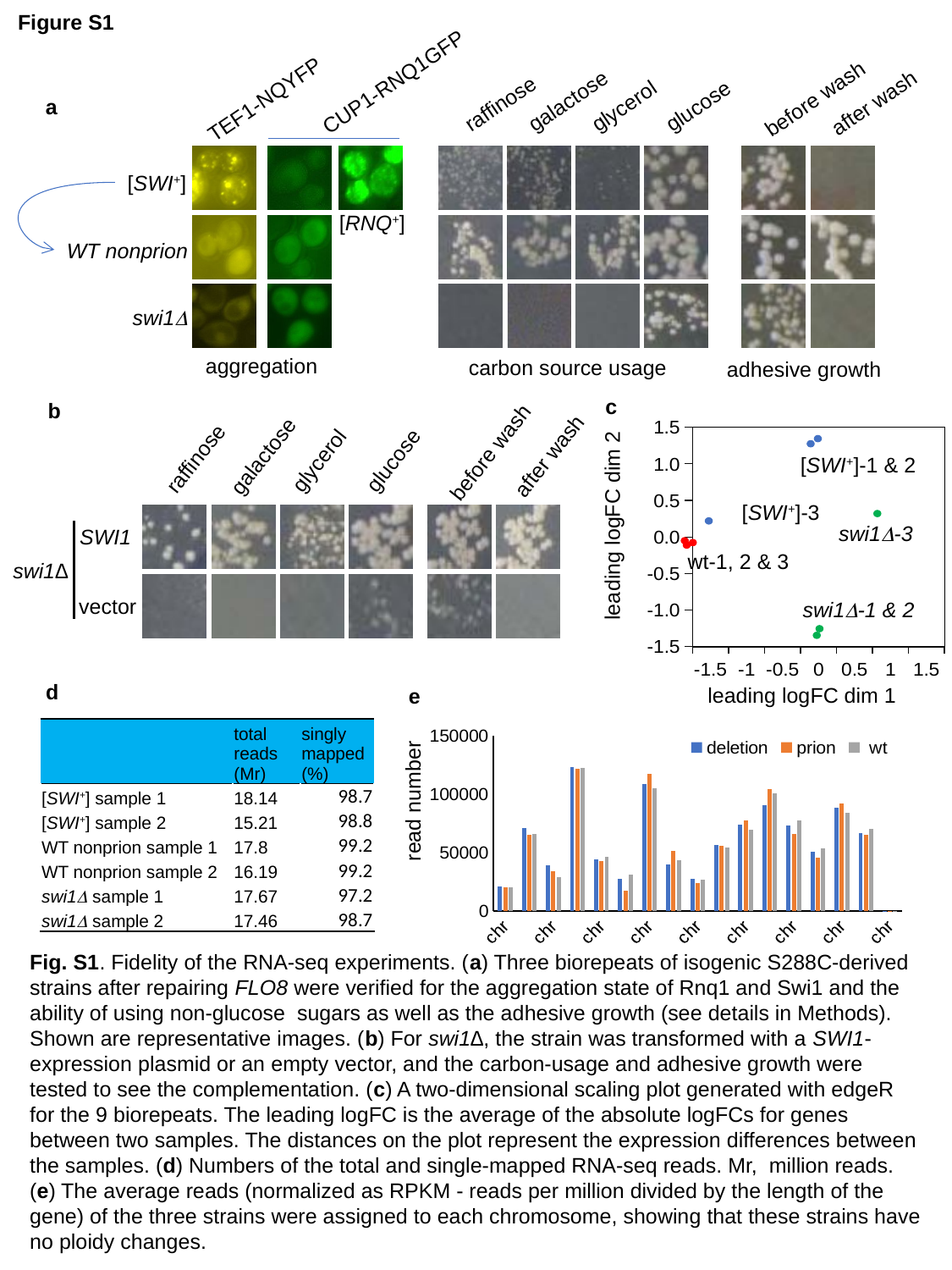

Figure S1
CUP1-RNQ1GFP
galactose
before wash
raffinose
TEF1-NQYFP
glycerol
glucose
after wash
a
[SWI+]
[RNQ+]
WT nonprion
swi1Δ
aggregation
carbon source usage
adhesive growth
c
### Chart
| Category | 1.5 | 1 | 0.5 | 0 | -0.5 | -1 | -1.5 |
|---|---|---|---|---|---|---|---|
| -1.5 | None | None | None | None | None | None | None |
| -1 | None | None | None | None | None | None | None |
| -0.5 | None | None | None | None | None | None | None |
| 0 | None | None | None | None | None | None | None |
| 0.5 | None | None | None | None | None | None | None |
| 1 | None | None | None | None | None | None | None |
| 1.5 | None | None | None | None | None | None | None |[SWI+]-1 & 2
[SWI+]-3
leading logFC dim 2
swi1Δ-3
wt-1, 2 & 3
swi1Δ-1 & 2
leading logFC dim 1
b
galactose
before wash
raffinose
glycerol
glucose
after wash
SWI1
swi1∆
vector
d
e
| | total reads (Mr) | singly mapped (%) |
| --- | --- | --- |
| [SWI+] sample 1 | 18.14 | 98.7 |
| [SWI+] sample 2 | 15.21 | 98.8 |
| WT nonprion sample 1 | 17.8 | 99.2 |
| WT nonprion sample 2 | 16.19 | 99.2 |
| swi1Δ sample 1 | 17.67 | 97.2 |
| swi1Δ sample 2 | 17.46 | 98.7 |
### Chart
| Category | deletion | prion | wt |
|---|---|---|---|
| chrI | 20781.773474105277 | 20533.85220729535 | 20264.501653970576 |
| chrII | 70759.15660252613 | 65085.5006798852 | 66002.7308657573 |
| chrIII | 38918.146355715835 | 34035.14620590408 | 28689.325197766553 |
| chrIV | 122965.31538155716 | 121479.03190336295 | 122510.03336066642 |
| chrV | 43984.85133231868 | 42404.90829496653 | 45977.731094969495 |
| chrVI | 27505.355771863968 | 17495.800477029254 | 31062.195941173115 |
| chrVII | 108248.77609409942 | 117425.63556153055 | 104619.18308539229 |
| chrVIII | 39424.66597946943 | 51292.41760899198 | 43627.53004034364 |
| chrIX | 27260.835392487174 | 23637.11982208182 | 27044.560064309582 |
| chrX | 56693.82451602342 | 55806.9679481816 | 54517.44638222526 |
| chrXI | 73604.33944624448 | 77671.3402012302 | 69060.95305453752 |
| chrXII | 90718.81428562013 | 103918.99939355401 | 100688.62871440036 |
| chrXIII | 73205.04236502512 | 65897.00721342003 | 77294.6550399596 |
| chrXIV | 50878.81251048803 | 45799.18507234291 | 53632.84650801485 |
| chrXV | 88354.36041711035 | 91790.595593544 | 84278.38906230913 |
| chrXVI | 66433.2632723677 | 65476.051286678165 | 70507.74461361377 |
| chrmt | 28.308190679103486 | 34.40919168801997 | 31.883087739005802 |read number
Fig. S1. Fidelity of the RNA-seq experiments. (a) Three biorepeats of isogenic S288C-derived strains after repairing FLO8 were verified for the aggregation state of Rnq1 and Swi1 and the ability of using non-glucose sugars as well as the adhesive growth (see details in Methods). Shown are representative images. (b) For swi1∆, the strain was transformed with a SWI1-expression plasmid or an empty vector, and the carbon-usage and adhesive growth were tested to see the complementation. (c) A two-dimensional scaling plot generated with edgeR for the 9 biorepeats. The leading logFC is the average of the absolute logFCs for genes between two samples. The distances on the plot represent the expression differences between the samples. (d) Numbers of the total and single-mapped RNA-seq reads. Mr, million reads. (e) The average reads (normalized as RPKM - reads per million divided by the length of the gene) of the three strains were assigned to each chromosome, showing that these strains have no ploidy changes.

## Slide 3
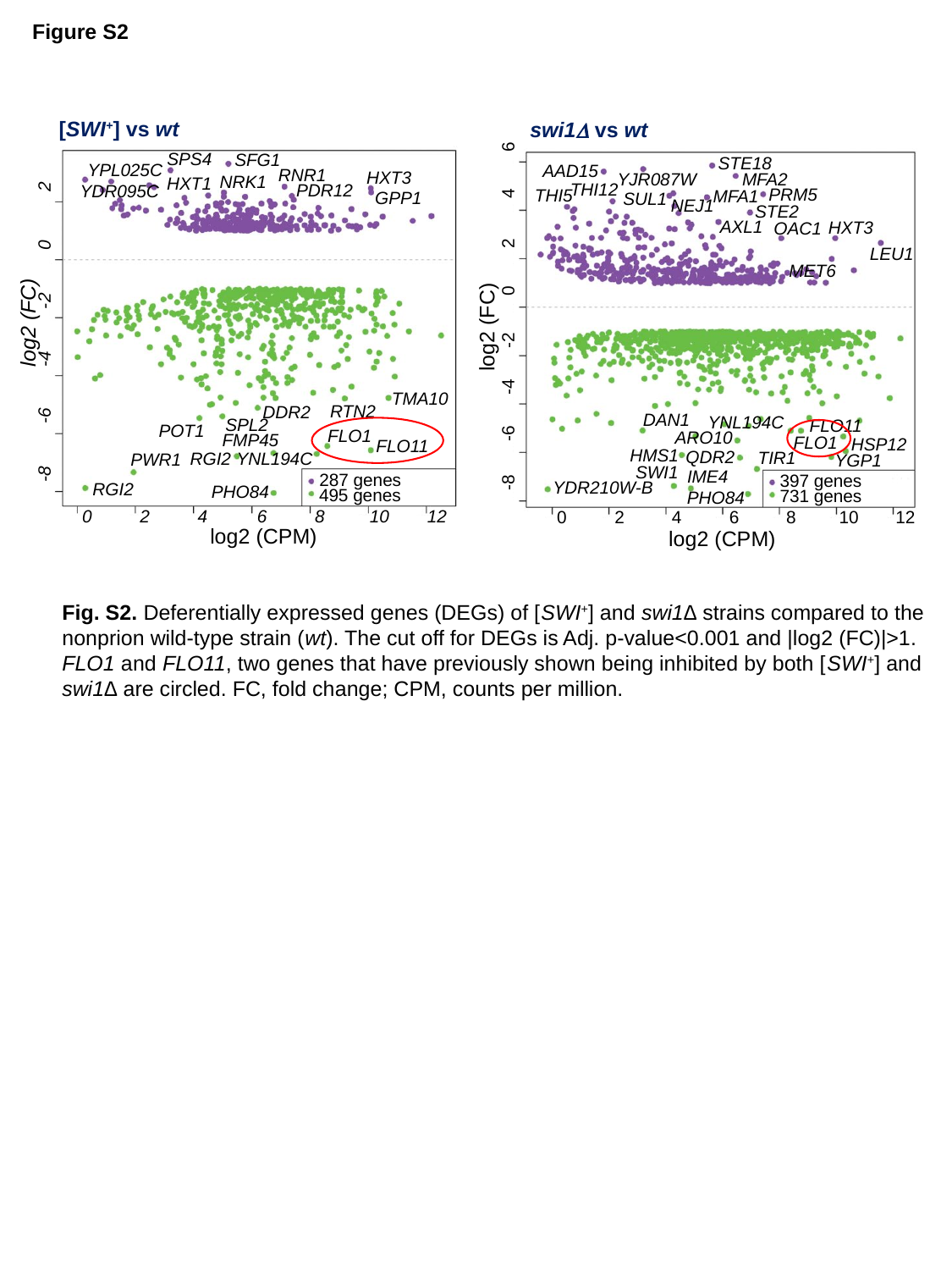

Figure S2
[SWI+] vs wt
SPS4
SFG1
YPL025C
RNR1
HXT3
NRK1
HXT1
PDR12
YDR095C
2
GPP1
0
log2 (FC)
-2
-4
TMA10
RTN2
DDR2
SPL2
-6
POT1
FLO1
FMP45
FLO11
RGI2
YNL194C
PWR1
287 genes
-8
RGI2
PHO84
495 genes
0
2
4
6
8
10
12
log2 (CPM)
swi1Δ vs wt
6
STE18
AAD15
YJR087W
MFA2
THI12
PRM5
THI5
MFA1
SUL1
4
NEJ1
STE2
AXL1
HXT3
OAC1
2
LEU1
MET6
0
log2 (FC)
-2
-4
DAN1
YNL194C
FLO11
ARO10
FLO1
-6
HSP12
HMS1
QDR2
TIR1
YGP1
SWI1
IME4
397 genes
YDR210W-B
-8
731 genes
PHO84
0
2
4
6
8
10
12
log2 (CPM)
Fig. S2. Deferentially expressed genes (DEGs) of [SWI+] and swi1∆ strains compared to the nonprion wild-type strain (wt). The cut off for DEGs is Adj. p-value<0.001 and |log2 (FC)|>1. FLO1 and FLO11, two genes that have previously shown being inhibited by both [SWI+] and swi1∆ are circled. FC, fold change; CPM, counts per million.

## Slide 4
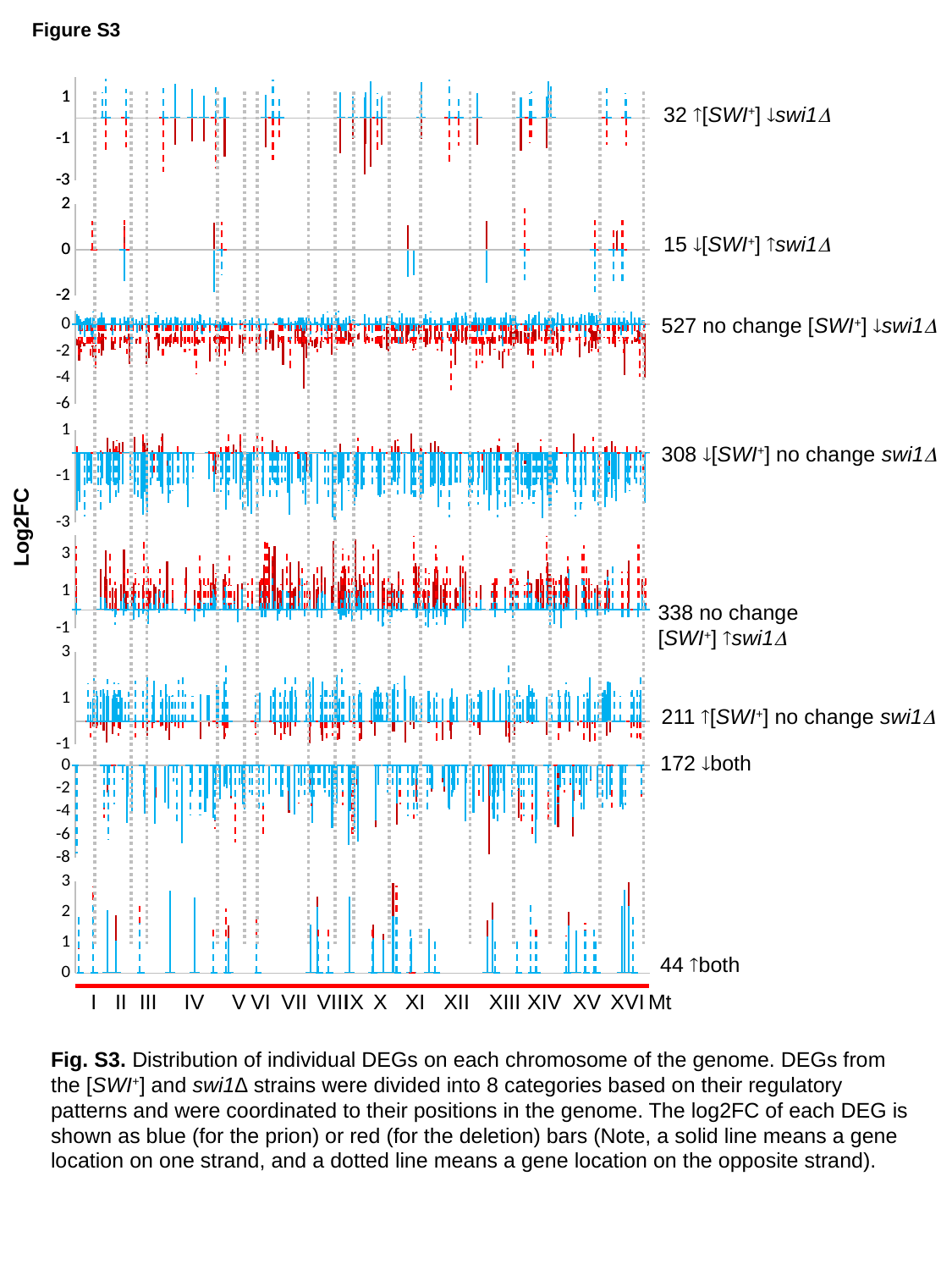

Figure S3
### Chart
| Category | [SWI+]log2FC | swi1 log2FC |
|---|---|---|
### Chart
| Category | [SWI+]log2FC | swi1 log2FC |
|---|---|---|32 [SWI+] swi1Δ
### Chart
| Category | [SWI+]log2FC | swi1 log2FC |
|---|---|---|
### Chart
| Category | [SWI+]log2FC | swi1 log2FC |
|---|---|---|15 [SWI+] swi1Δ
### Chart
| Category | [SWI+]log2FC | swi1 log2FC |
|---|---|---|
### Chart
| Category | [SWI+]log2FC | swi1 log2FC |
|---|---|---|527 no change [SWI+] swi1Δ
### Chart
| Category | [SWI+]log2FC | swi1 log2FC |
|---|---|---|
### Chart
| Category | [SWI+]log2FC | swi1 log2FC |
|---|---|---|308 [SWI+] no change swi1Δ
Log2FC
### Chart
| Category | [SWI+]log2FC | swi1 log2FC |
|---|---|---|
### Chart
| Category | [SWI+]log2FC | swi1 log2FC |
|---|---|---|338 no change [SWI+] swi1Δ
### Chart
| Category | [SWI+]log2FC | swi1 log2FC |
|---|---|---|
### Chart
| Category | [SWI+]log2FC | swi1 log2FC |
|---|---|---|211 [SWI+] no change swi1Δ
172 both
### Chart
| Category | [SWI+]log2FC | swi1 log2FC |
|---|---|---|
### Chart
| Category | [SWI+]log2FC | swi1 log2FC |
|---|---|---|
### Chart
| Category | [SWI+]log2FC | swi1 log2FC |
|---|---|---|
### Chart
| Category | [SWI+]log2FC | swi1 log2FC |
|---|---|---|44 both
I
II
III
IV
V
VI
VII
VIII
IX
X
XI
XII
XIII
XIV
XV
XVI
Mt
Fig. S3. Distribution of individual DEGs on each chromosome of the genome. DEGs from the [SWI+] and swi1∆ strains were divided into 8 categories based on their regulatory patterns and were coordinated to their positions in the genome. The log2FC of each DEG is shown as blue (for the prion) or red (for the deletion) bars (Note, a solid line means a gene location on one strand, and a dotted line means a gene location on the opposite strand).

## Slide 5
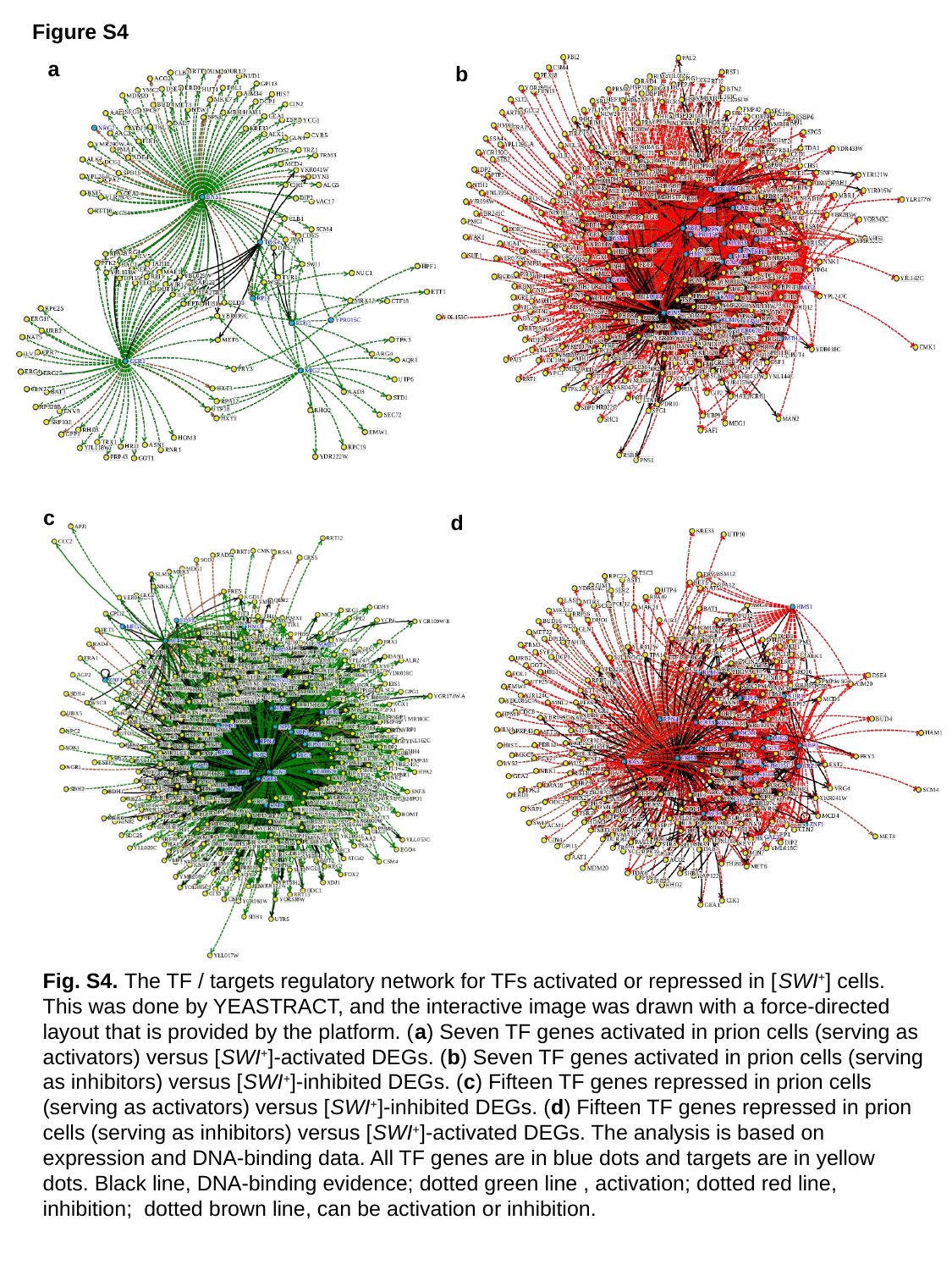

Figure S4
a
b
c
d
Fig. S4. The TF / targets regulatory network for TFs activated or repressed in [SWI+] cells. This was done by YEASTRACT, and the interactive image was drawn with a force-directed layout that is provided by the platform. (a) Seven TF genes activated in prion cells (serving as activators) versus [SWI+]-activated DEGs. (b) Seven TF genes activated in prion cells (serving as inhibitors) versus [SWI+]-inhibited DEGs. (c) Fifteen TF genes repressed in prion cells (serving as activators) versus [SWI+]-inhibited DEGs. (d) Fifteen TF genes repressed in prion cells (serving as inhibitors) versus [SWI+]-activated DEGs. The analysis is based on expression and DNA-binding data. All TF genes are in blue dots and targets are in yellow dots. Black line, DNA-binding evidence; dotted green line , activation; dotted red line, inhibition; dotted brown line, can be activation or inhibition.

## Slide 6
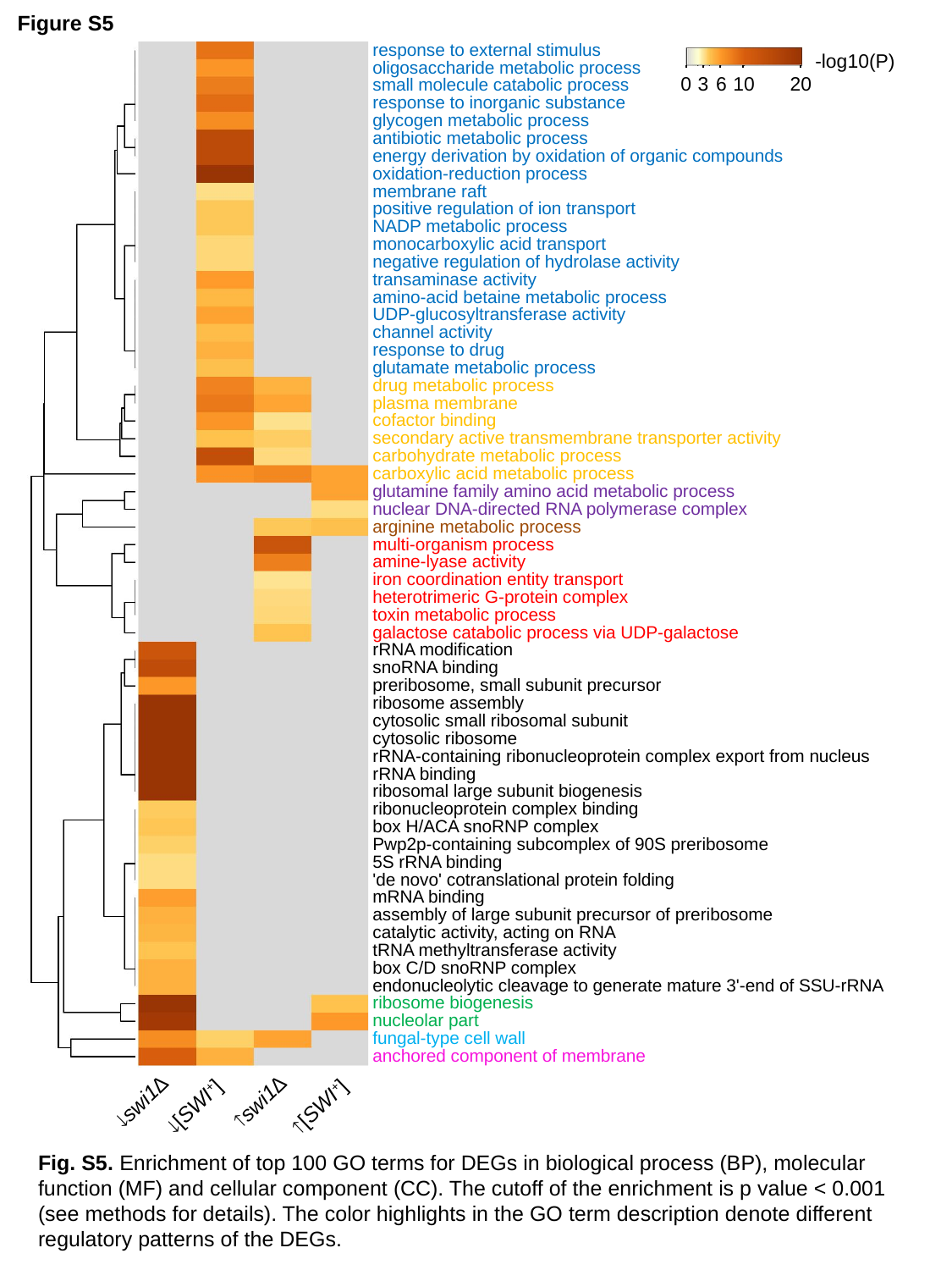

Figure S5
response to external stimulus
oligosaccharide metabolic process
small molecule catabolic process
response to inorganic substance
glycogen metabolic process
antibiotic metabolic process
energy derivation by oxidation of organic compounds
oxidation-reduction process
membrane raft
positive regulation of ion transport
NADP metabolic process
monocarboxylic acid transport
negative regulation of hydrolase activity
transaminase activity
amino-acid betaine metabolic process
UDP-glucosyltransferase activity
channel activity
response to drug
glutamate metabolic process
drug metabolic process
plasma membrane
cofactor binding
secondary active transmembrane transporter activity
carbohydrate metabolic process
carboxylic acid metabolic process
glutamine family amino acid metabolic process
nuclear DNA-directed RNA polymerase complex
arginine metabolic process
multi-organism process
amine-lyase activity
iron coordination entity transport
heterotrimeric G-protein complex
toxin metabolic process
galactose catabolic process via UDP-galactose
rRNA modification
snoRNA binding
preribosome, small subunit precursor
ribosome assembly
cytosolic small ribosomal subunit
cytosolic ribosome
rRNA-containing ribonucleoprotein complex export from nucleus
rRNA binding
ribosomal large subunit biogenesis
ribonucleoprotein complex binding
box H/ACA snoRNP complex
Pwp2p-containing subcomplex of 90S preribosome
5S rRNA binding
'de novo' cotranslational protein folding
mRNA binding
assembly of large subunit precursor of preribosome
catalytic activity, acting on RNA
tRNA methyltransferase activity
box C/D snoRNP complex
endonucleolytic cleavage to generate mature 3'-end of SSU-rRNA
ribosome biogenesis
nucleolar part
fungal-type cell wall
anchored component of membrane
-log10(P)
3
0
6
10
20
swi1∆
swi1∆
[SWI+]
[SWI+]
Fig. S5. Enrichment of top 100 GO terms for DEGs in biological process (BP), molecular function (MF) and cellular component (CC). The cutoff of the enrichment is p value < 0.001 (see methods for details). The color highlights in the GO term description denote different regulatory patterns of the DEGs.
